# Multimodal Cataract Severity Classification from AS-OCT Examination Reports: Analyzing the Complementarity of B-Scan Images and A-Scan Profiles

**DOI:** 10.3390/diagnostics16203362

**Published:** 2026-10-15

**Authors:** Min-Seok Kim, Gang-Min Park, Ho-Gil Jung, Jong-Joo Lee

**Affiliations:** 1 Department of Data Science, Seoul National University of Science and Technology, Seoul 01811, Republic of Korea; mskim2659@seoultech.ac.kr (M.-S.K.); gangmin.park@seoultech.ac.kr (G.-M.P.); 2 Department of Ophthalmology, National Medical Center, Seoul 04564, Republic of Korea

**Keywords:** cataract severity assessment, anterior segment optical coherence tomography (AS-OCT), multimodal learning, A-scan profile, cross-modal attention

## Abstract

**Background/Objectives:** Deep learning on anterior segment optical coherence tomography (AS-OCT) has been applied mainly to nuclear cataract. This study evaluated whether the A-scan profile in Anterion reports adds information to the B-scan image for classifying nuclear (N), cortical (C), and posterior subcapsular (P) cataract severity. **Methods:** B-scan images and A-scan profiles were extracted from 1416 reports of 691 patients. LOCS III grades were converted into three levels (None/Mild, Moderate, Advanced); inter-observer agreement was excellent (weighted κ 0.84–0.97). Image and profile encoders were combined using five fusion strategies and evaluated with patient-level 5-fold cross-validation. **Results:** Adding the A-scan profile improved F1 for N (+0.030) and P (+0.094) but not for C (−0.002). All fusion strategies outperformed single-modality models and performed comparably (average F1 0.514–0.531). For P, the gain was largest at the Moderate grade (F1 0.183 to 0.336), and masking the profile segment containing the posterior capsule caused the largest drop. B-scan line profiles could not replace the device-provided profile, and most misclassifications occurred between adjacent grades. **Conclusions:** The A-scan profile provided a useful incremental contribution, particularly for N and P, in this single-center dataset. The approach may be useful in retrospective and multi-center settings, but this remains to be demonstrated.

## 1. Introduction

Cataract is a condition in which the crystalline lens loses its transparency, and it accounts for approximately 45% of global blindness, making it the leading cause of preventable vision loss [1,2]. The burden continues to grow with population aging. Cataracts are classified into three types according to the location of opacity. Nuclear cataract (N) involves progressive hardening and yellow-brown discoloration of the central nucleus, inducing a myopic shift and advancing gradually over years. Cortical cataract (C) presents as radial or wedge-shaped opacities that progress from the lens periphery toward the center, causing glare and peripheral visual disturbance. Posterior subcapsular cataract (P) forms as a thin plaque opacity just anterior to the posterior capsule; because it lies near the visual axis, it reduces near vision and photopic acuity relatively early [3]. In clinical practice, the three types frequently coexist as mixed cataract rather than appearing in isolation, and each differs in progression rate and impact on visual function. Although the decision and timing of cataract surgery depend primarily on visual acuity, glare, contrast sensitivity, symptoms, and functional limitation rather than on LOCS III severity alone—particularly for P, in which a relatively small opacity can be markedly symptomatic—type-specific severity assessment provides complementary information for monitoring progression and supporting clinical evaluation.

The reference standard for cataract severity assessment is the Lens Opacities Classification System III (LOCS III), in which an examiner compares slit-lamp findings against standardized reference photographs to assign a grade for each type [4]. However, as this process relies on subjective visual comparison, inter- and intra-observer variability is inevitable, which limits its utility in longitudinal follow-up and multi-center studies [5]. An examiner-independent, quantitative assessment tool is therefore needed. Unlike slit-lamp-based evaluation, anterior segment optical coherence tomography (AS-OCT) captures a full cross-sectional image of the lens non-invasively, depicting the nuclear, cortical, and posterior subcapsular regions within a single scan. Its lower sensitivity to examination conditions compared with slit-lamp photography enables reproducible quantitative assessment [6]. These properties have motivated a growing body of work applying deep learning to automatic grading from AS-OCT images [7,8,9,10].

These studies, however, commonly assume access to the raw data acquired by the imaging device. In practice, clinical imaging data in ophthalmology are often stored in proprietary formats specific to each manufacturer, and retrieval from institutional archiving systems can be constrained by interoperability limitations [11,12]. Public datasets similarly tend to provide display-rendered images rather than raw measurements [13]. In AS-OCT examinations as well, only the examination report, rather than the original scan data, may be available, particularly in retrospective studies and multi-center data collection. The cross-sectional images in these reports are display-oriented outputs whose brightness and contrast have been adjusted for clinical readability and onto which anatomical boundary markers are overlaid; consequently, pixel intensity cannot be assumed to reflect tissue optical density on a consistent scale. In other words, although the anatomical structure of the lens can be observed from the report, obtaining density-related information directly from image intensity is unreliable.

This study addresses this limitation by exploiting an additional representation contained within the same examination report. The Anterion AS-OCT examination report [14] includes, alongside the two-dimensional cross-sectional image (B-scan), a separate one-dimensional reflectance intensity profile along the visual axis (A-scan profile). The two representations originate from the same swept-source interferometric measurement but undergo independent post-processing pathways and are rendered in different forms. The B-scan image provides broad spatial coverage encompassing the nuclear, cortical, and posterior subcapsular regions, yet the non-linear contrast adjustments applied for clinical readability compromise the physical linearity of pixel intensity. The A-scan profile, by contrast, undergoes detector-based normalization that better preserves signal intensity characteristics relative to the display-adjusted B-scan image, enabling more consistent cross-sample comparison, although residual per-scan gain variations may remain. Its spatial coverage, however, is confined to the one-dimensional region traversed by the visual axis. Given these complementary properties, nuclear cataract (N), which directly overlaps with the visual axis, and posterior subcapsular cataract (P), which lies near the axis, are expected to be captured more effectively through the axial intensity variations in the A-scan profile. Cortical cataract (C), in contrast, extends in a scattered and asymmetrical pattern toward the lens periphery away from the visual axis, suggesting that the two-dimensional spatial information of the B-scan image is more important for its assessment. The geometric relationship between opacity location and the visual axis thus suggests that the two modalities provide complementary information that can improve classification performance across cataract types. Building on this reasoning, this study constructs a multimodal cataract severity classification system using Anterion examination reports. B-scan images and A-scan profiles are automatically extracted from the reports and encoded by a pretrained Vision Transformer and a one-dimensional residual network, respectively; the fused features are then used to simultaneously predict the severity of N, C, and P. To our knowledge, this is the first cataract classification study to combine AS-OCT cross-sectional images and axial intensity profiles as complementary modalities. The contributions of this study are summarized as follows.

1. We present a multimodal approach that combines the B-scan image and A-scan profile from a standard AS-OCT examination report without requiring raw device data, and compare five fusion strategies to analyze the signal modality’s contribution to each cataract type, with consistent gains for P across all fusion strategies.
2. We show that the A-scan profile’s contribution depends on the location of opacity relative to the visual axis: improvement for N and P, whose opacities lie along the visual axis (F1 +0.030 and +0.094), but not for peripherally distributed C (−0.002). Region-wise masking and extraction pathway comparison further support the selective and non-redundant nature of this contribution.
3. Unlike prior studies limited to nuclear grading or presence–absence detection for C and P [7,8,9,15], this study classifies all three types into three severity grades, providing a broader basis for cross-type analysis of modality contributions.

## 2. Related Work

### 2.1. Imaging Modalities for Automated Cataract Grading

Automated approaches to cataract assessment differ according to the imaging modality employed. Fundus photography is suited to large-scale screening but reflects lens opacity only indirectly and cannot distinguish among cataract types [16]. Slit-lamp and retro-illumination photographs observe the lens directly, enabling type-specific evaluation. Son et al. [17] reported a rare instance in which a CNN ensemble applied to slit-lamp and retro-illumination images assessed all three types (N, C, P). These modalities, however, are two-dimensional projections that cannot capture the axial density distribution within the lens, and they are sensitive to illumination angle and pupil dilation. Quantitative assessment of cataract types, which are defined by the location of opacity, requires a modality capable of cross-sectional observation of the lens interior; AS-OCT meets this requirement.

### 2.2. AS-OCT-Based Cataract Grading

The validity of quantifying lens opacity with AS-OCT has been established in clinical studies. Wong et al. [18] reported that AS-OCT-derived optical density correlated significantly with LOCS III grades, and Wang et al. [19] confirmed that objective quantification of nuclear opacity is feasible with swept-source AS-OCT. These studies, however, remained at the level of correlation analysis and did not proceed to automatic grading.

Subsequent deep-learning efforts have centered on the difficulty of distinguishing adjacent grades. GraNet [7], the first attempt at AS-OCT-based automatic grading, achieved only 57.86% on six-grade nuclear classification, exposing the challenge of the task. Later studies addressed this problem through different strategies: region-based attention incorporating clinical prior knowledge [20], a ranking-based approach that decomposes multi-class grading into binary comparisons [8], and ordinal regression that preserves inter-grade continuity [9]. All of these, however, target nuclear cataract alone, and studies addressing cortical cataract remain scarce [21].

Approaches that evaluate all three types have emerged only recently. Takinami et al. [15] employed a self-supervised Vision Transformer for N, C, and P, yet graded only nuclear cataract at multiple levels (2-, 3-, 6-class) while limiting cortical and posterior subcapsular assessment to presence–absence detection. Hu et al. [22] performed LOCS III grading for all three types through a mask-guided architecture that exploits lens substructure segmentation as spatial prior, which required per-image pixel-level segmentation annotations. Despite differences in scope and approach, all of the above studies share the common assumption of access to raw device-acquired images.

### 2.3. Recovering Numerical Information from Rendered Records

When raw measurement data are unavailable, recovering numerical information from rendered outputs has been explored in several medical domains. The most extensive body of work exists in electrocardiography, where a large fraction of historical recordings survive only as paper printouts. Automated tools have been developed to extract waveform time series from scanned ECG images, and the 2024 PhysioNet Challenge jointly addressed printed-ECG digitization and cardiac disease classification [23]. A parallel effort has recently emerged in audiology: audiograms are routinely stored as chart images in electronic medical records, and deep-learning pipelines now digitize frequency-threshold values from these images, achieving processing speeds an order of magnitude faster than manual extraction [24]. In ophthalmology, Mun et al. [25] developed software that automatically extracts numerical measurements and tomographic images from OCT examination report files and demonstrated its application to over 430,000 reports from a single tertiary hospital, converting them into a structured database. This study follows the same rationale of recovering numerical signals from rendered examination outputs. Unlike the above examples, however, the recovered profile is not used in isolation but is combined with the cross-sectional image from the same report as a complementary modality.

### 2.4. Multimodal Fusion in Ophthalmic Imaging

Multimodal studies in ophthalmology have combined outputs from different examinations, such as fundus photography, OCT, OCTA, and visual field testing, for diagnosis and prognosis of glaucoma, diabetic retinopathy, and age-related macular degeneration [26,27]. Fusion strategies are broadly categorized into feature-level, decision-level, and hybrid-level approaches. Recent work has increasingly adopted attention-based inter-modal interaction: Liu et al. [28] introduced a cross-modal attention module that queries spatially corresponding features across fundus and OCT images for retinal disease classification, and Zhou et al. [29] proposed a transformer-based alignment-and-fusion framework for multi-modal glaucoma recognition. In all of these cases, however, the modalities being combined are two-dimensional (or three-dimensional) images of the posterior segment. To our knowledge, no prior study has fused a two-dimensional cross-sectional image with a one-dimensional axial intensity profile. Because the two representations differ in spatial dimensionality, the effectiveness of their fusion and the pattern of each modality’s contribution cannot be assumed from existing results and require empirical verification. Existing multimodal studies have also given limited attention to how fusion strategies and per-modality contributions vary across disease subtypes, which is a central question of this work.

## 3. Materials and Methods

This section describes the data processing pipeline and model architecture of the proposed framework. Section 3.1 presents the structure of the AS-OCT examination report and the dataset used in this study. Section 3.2 describes the procedures for extracting the B-scan image and A-scan intensity profile from the report. Section 3.3 defines the criteria for converting LOCS III grades into three severity levels, and Section 3.4 describes the model architecture including the encoding pipelines and fusion strategies.

### 3.1. Data Source

#### 3.1.1. AS-OCT Examination and Report Structure

The data used in this study were obtained from anterior segment examination reports acquired with the Heidelberg Anterion (Heidelberg Engineering, Heidelberg, Germany), a swept-source AS-OCT device. The Anterion uses a 1300 nm light source in a non-contact configuration, acquiring 50,000 A-scans per second with an axial resolution below 10 µm and a scan depth of 14±0.5 mm [30,31]. Examinations were performed using the Cataract App (review module v1.4.4.0, data acquisition module v1.4.4.0) on the HEYEX 2 platform (v2.5.5.1950).

An A-scan (axial scan) is the fundamental unit of one-dimensional depth-resolved measurement. The raw interference signal collected by the hardware detector is converted into axial reflectance data through *k*-space calibration, which compensates for the nonlinear wavelength sweep of the light source, followed by fast Fourier transform (FFT). These A-scan data constitute the one-dimensional reflectance characteristics of the anterior segment (A-scan profile) and also serve as the building blocks of two-dimensional B-scan images through lateral scanning.

**A-scan (Axial scan) profile**: The A-scan profile is generated through a detector-based normalization pathway that is separate from the display adjustments applied to the B-scan image. The system derives an axial reflectivity profile based on the A-scan data along the device’s optical axis [14], with measurements centered on the corneal vertex by eye tracking while the patient fixates on the built-in central fixation target [31]; this axis thus approximates the visual axis and is hereafter referred to as the visual axis. The profile is normalized to the dynamic range of the detector according to the manufacturer’s proprietary algorithm.

The resulting signal is expressed on an arbitrary-unit (a.u.) scale and rendered as a one-dimensional graph in the examination report. This graph is referred to as the A-scan profile throughout this paper.

**B-scan (Brightness scan) image**: The B-scan image is produced by combining multiple laterally acquired A-scan lines and applying geometric post-processing for visualization [14]. Up to 1024 reconstructed A-scan lines are arranged sequentially to form a two-dimensional frame. Eye-tracking-assisted averaging of up to eight B-scan frames suppresses speckle noise [14], after which non-linear contrast adjustment is applied to enhance the visibility of anatomical boundaries. The resulting cross-sectional image is rendered in the report for clinical interpretation.

**Figure 1:**
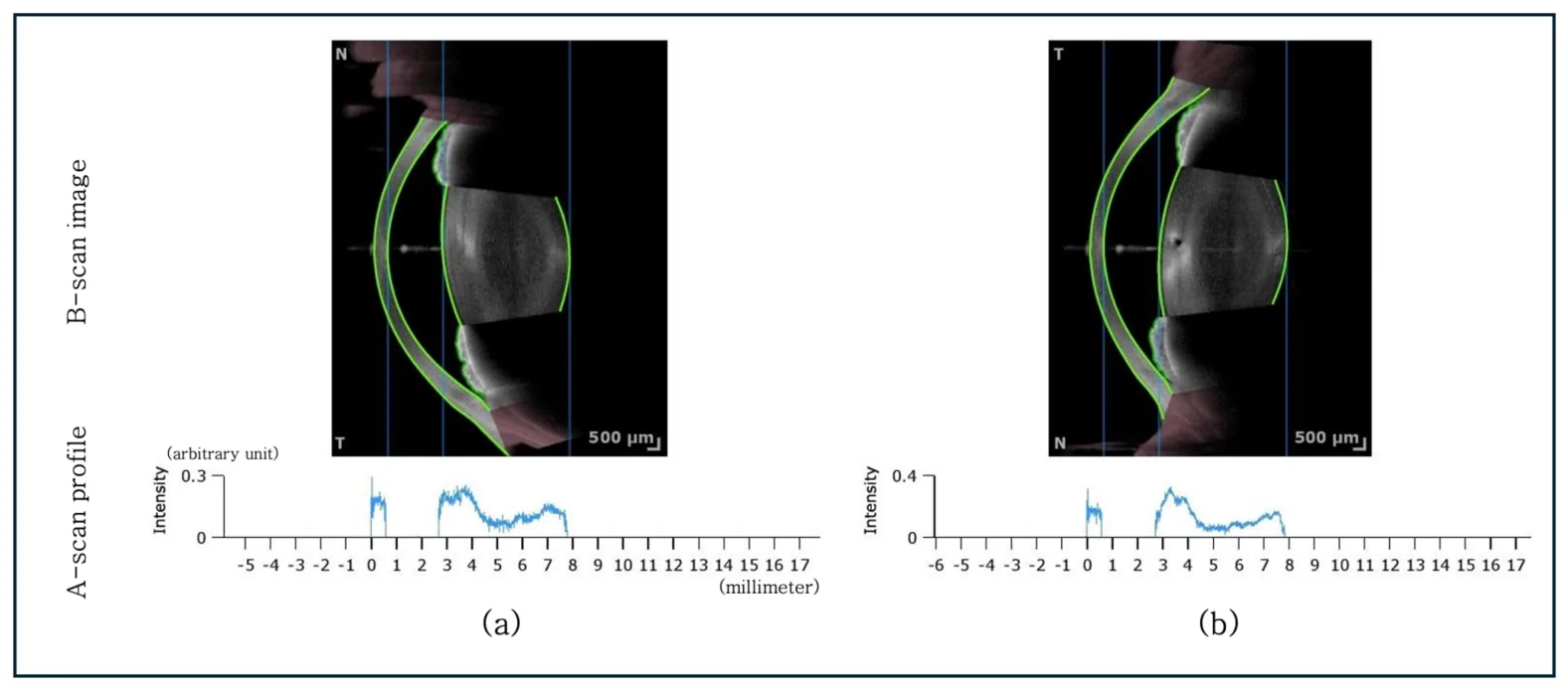
B-scan and A-scan profile layout in the Anterion AS-OCT examination report. (**a**) Right eye (oculus dexter, OD) and (**b**) left eye (oculus sinister, OS) B-scan cross-sectional images with corresponding A-scan profiles. The horizontal line on each B-scan indicates the visual axis along which the A-scan profile is acquired.

Figure 1 shows the layout of the B-scan and A-scan profile regions in an Anterion AS-OCT examination report. The report contains the two-dimensional B-scan image depicting the spatial structure of the lens alongside the one-dimensional A-scan profile representing the reflectance intensity along the visual axis. The horizontal axis of the graph corresponds to physical depth (mm) and the vertical axis to signal intensity; the *y*-axis range adjusts automatically according to signal amplitude, but the axis scale printed on the report can be used to convert all samples to a common intensity scale. This study extracts these two representations from the report and uses them as inputs to a multimodal model.

#### 3.1.2. Study Population and Data Collection

Data were collected from cataract patients at the National Medical Center (Seoul, Republic of Korea) between June 2021 and October 2025. The study was approved by the institutional review board (NMC IRB 2025-09-030-002). Reports for the right eye (oculus dexter, OD) and left eye (oculus sinister, OS) were obtained from each examination.

Two experienced board-certified ophthalmologists divided the dataset between them, and each independently graded their assigned subset according to LOCS III criteria for N, C, and P; of the 1416 examinations included in the final dataset, they graded 1211 and 205, respectively. LOCS III grades were assigned retrospectively from stored slit-lamp and retro-illumination photographs acquired after pharmacological pupil dilation at routine clinic visits, which were conducted separately from the Anterion examination. For each Anterion examination, the grade from the clinic visit closest in date to the Anterion acquisition was used as the reference label. Among the 1416 examinations included in the final analysis, the slit-lamp examination and the Anterion examination were performed on the same day in 79.0% and within 30 days in 93.5%; when performed on different days, the median interval was 23 days (interquartile range, 14–35 days). To assess the reliability of the reference grades, an additional ophthalmologist who had not participated in the original grading independently re-graded a subset of 271 visits from 163 patients using the same stored photographs, according to the same LOCS III criteria, without access to the original grades. Agreement was quantified for each cataract type using the quadratic-weighted Cohen’s κ for the three-level severity grades used as training targets and the intraclass correlation coefficient (ICC; two-way random effects, absolute agreement, single rater) for the original LOCS III scores, computed over visits in which both graders recorded the respective type. Inter-observer agreement was excellent for all three types (weighted κ: N 0.84, C 0.92, P 0.97; ICC: N 0.92, C 0.98, P 0.99; Table 1).

Of the initial 1783 examinations from 726 patients, 130 were excluded because a usable lens region could not be obtained from the B-scan image; in 98 of these the A-scan profile was nevertheless extracted successfully from the same report, whereas in the remaining 32 the optical character recognition (OCR)-based scale detection of the A-scan graph also failed. Pseudophakic eyes with an intraocular lens (IOL) had already been excluded at the labeling stage, because LOCS III grades are not recorded for such eyes. The 130 excluded examinations had a higher proportion of Advanced grades than the final dataset (N 6.2% vs. 2.5%; P 15.4% vs. 8.1%), suggesting that lens region extraction failed more often in denser cataracts. An additional 11 examinations in which the cropped B-scan height or width was below 80 pixels and 1 examination in which the valid A-scan profile length was below 50 points were also excluded to avoid excessive upsampling relative to the target input resolution. These thresholds were set empirically, and only 12 examinations were affected. Of the remaining 1641 examinations, 225 were duplicates in which the same scan had been saved more than once, with an identical B-scan image and A-scan profile; these were removed, retaining a single copy. The final dataset comprised 1416 examinations of 1155 eyes from 691 patients. Of these patients, 464 contributed both eyes and 519 contributed more than one examination; 205 eyes underwent repeat examinations. The repeat examinations comprised 94 additional same-day acquisitions and follow-up examinations on different dates in 151 eyes of 116 patients, yielding 1322 unique eye-visits.

### 3.2. Data Preprocessing

**Figure 2:**
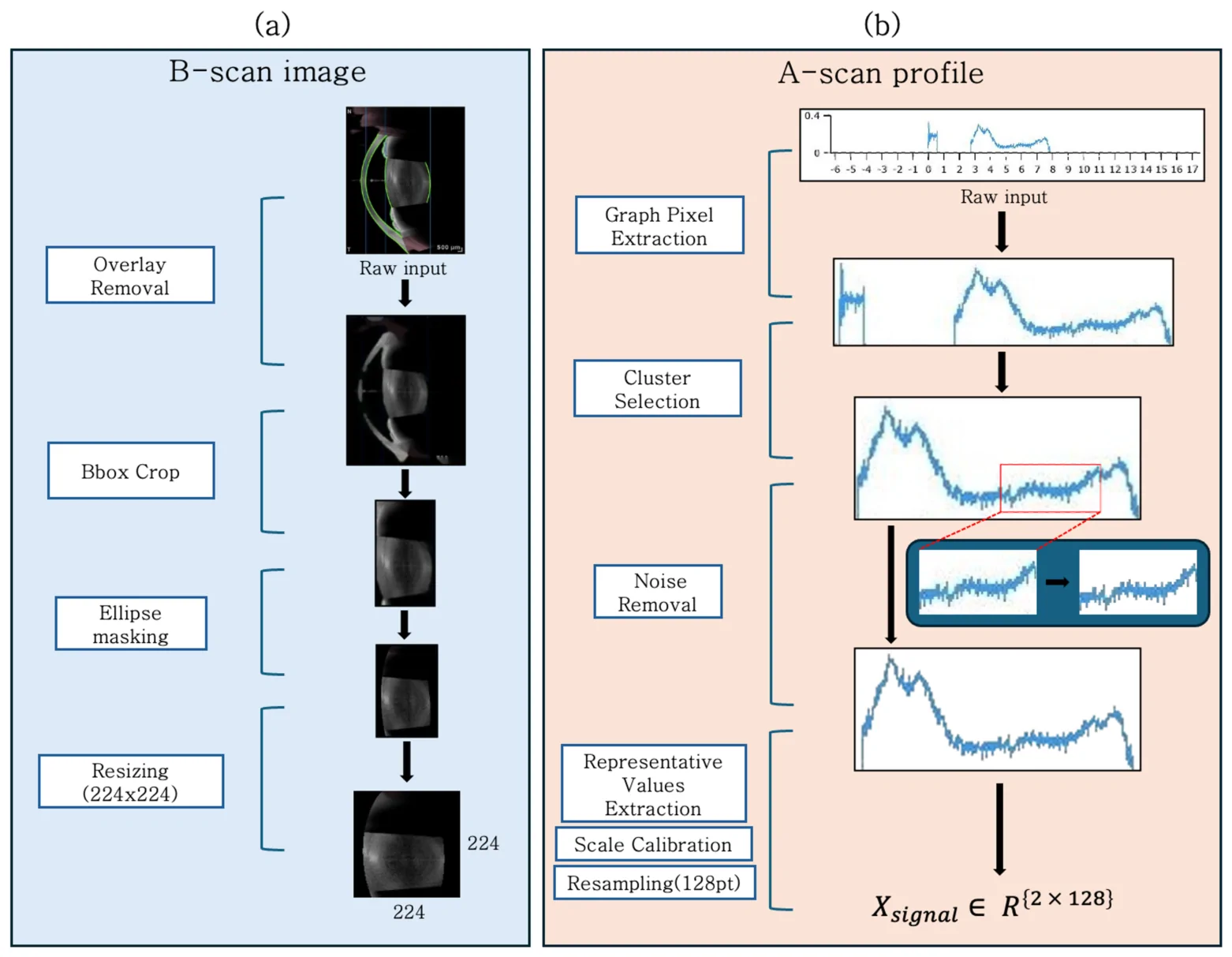
Data preprocessing pipeline from the examination report. (**a**) B-scan image preprocessing. (**b**) A-scan profile preprocessing.

Because the examination report contains multiple examination items within a single document, the B-scan image and A-scan profile are first localized and separated using their fixed coordinate regions. Each modality is then subjected to a dedicated preprocessing pipeline to produce the final model inputs. The overall preprocessing workflow is illustrated in Figure 2.

#### 3.2.1. B-Scan Image Preprocessing

Isolating the lens region from the report image proceeds in three steps: overlay removal, lens region cropping, and ellipse masking.

**Overlay removal and lens region cropping**: The B-scan in the report contains device-generated anatomical boundary markers (green contour lines) and text annotations overlaid on the image. Color overlay regions are first detected in HSV color space using saturation and value thresholds (saturation >25, value >35) and restored via TELEA inpainting [32]. Text annotation regions are removed in the same manner. The green boundary lines (HSV hue 30–90°, saturation >40) are then detected separately to determine the separation between the cornea and the lens. On the overlay-free grayscale image, binarization followed by morphological closing and opening is applied to extract connected components, and the component corresponding to the lens is selected based on proximity to the image center and area. The detected lens region is cropped with 5% padding around its bounding box.

**Ellipse masking and resizing**: An ellipse is fitted to the combined set of lens tissue coordinates and green boundary coordinates within the cropped region to approximate the lens shape. The fitted ellipse is expanded by 5% along both axes, and pixels outside the expanded ellipse are set to zero to remove extralenticular structures such as the iris and cornea. The resulting image is resized to 224×224 and normalized using ImageNet mean and standard deviation.

#### 3.2.2. A-Scan Profile Preprocessing

The following procedure recovers a one-dimensional numerical profile from the rendered A-scan graph in the report.

**Graph pixel extraction**: The set of pixels S corresponding to the graph curve is extracted using a color filter:

(1) S={(x,y)∣R(x,y)<180,G(x,y)>110,B(x,y)>110},

where R(x,y), G(x,y), and B(x,y) are the RGB channel values at pixel (x,y). Connected-component analysis is then applied, and components with a vertical extent below 2 pixels are discarded as grid lines or rendering artifacts.

**Cluster selection**: When consecutive missing columns span 30 pixels or more, the profile is split into separate clusters, and the cluster containing the most valid pixels is selected. The A-scan intensity profile typically consists of two clusters: an anterior peak from corneal reflection and a lens segment. The wide gap corresponding to the anterior chamber separates them automatically under the 30-pixel threshold. Because the lens segment spans the full axial extent from the anterior to the posterior capsule, it is always longer than the corneal peak, and the largest-cluster rule reliably isolates the lens region.

**Noise removal**: Residual pixels near the curve boundary may persist within the selected cluster. For each column, vertically contiguous pixel groups are identified; groups shorter than 2 pixels are treated as residual noise and removed. The rendered curve has a consistent line width and therefore exhibits at least 2 pixels of vertical continuity, whereas isolated residual pixels do not satisfy this condition. This step also removes sporadic pixels that appear within a column but are separated from the main curve body.

**Column-wise representative values**: Because the curve is rendered with a finite line width, multiple pixels are detected per column. Let $D_{x}=\left\{d_{1},\ldots ,d_{n}\right\}$ denote the set of distances from the *x*-axis for column *x*. The representative value and spread are defined as

(2) $$y_{\mathrm{mean}}\left(x\right)=\frac{1}{n}\sum_{i=1}^{n}d_{i},\;y_{\mathrm{std}}\left(x\right)=\sqrt{\frac{1}{n}\sum_{i=1}^{n}{\left(d_{i}-y_{\mathrm{mean}}\left(x\right)\right)}^{2}}.$$

$y_{\mathrm{mean}}$ corresponds to the intensity value at that position. $y_{\mathrm{std}}$ reflects the vertical spread of the detected curve pixels, which is jointly determined by the line width and the local slope of the curve; it therefore carries auxiliary information about the local rate of intensity change.

**Scale calibration**: The maximum *y*-axis scale label is detected via OCR (Tesseract 5.2.0) convert pixel-domain distances to calibrated intensity values (a.u.):

(3) $$d_{\mathrm{cal}}=d_{\mathrm{pixel}}\times \frac{s}{y_{\mathrm{axis}}},$$

where $d_{\mathrm{pixel}}$ is the pixel-domain distance, *s* is the detected maximum *y*-axis scale, and $y_{\mathrm{axis}}$ is the pixel distance between the *x*-axis and the maximum *y*-axis label. The axis scales observed in this dataset were predominantly 0.3 or 0.4; a small number of unlabeled examinations exhibited other scales (e.g., 0.5) but lacked grade labels and were therefore excluded from the analysis. OCR-based scale detection was applied to all 1783 examinations, yielding successful detection in 1701 cases (95.4%). Visual comparison against the original graphs for a random subset of 500 cases confirmed 100% detection accuracy. Of the 82 failed cases, 50 were manually corrected, and the remaining 32 had already been excluded due to B-scan lens detection failure (Section 3.2.1).

**Resampling**: Raw profiles of varying lengths are resampled to 128 points via linear interpolation. Each sample is thus represented as a two-channel profile:

(4) $$X_{\mathrm{signal}}\in R^{2\times 128},$$

with $y_{\mathrm{mean}}$ and $y_{\mathrm{std}}$ as channels. All thresholds used in the two preprocessing pipelines above were set empirically through preliminary inspection of the training data.

### 3.3. Severity Label Design

Cataract grades in the dataset follow the LOCS III system, recorded at 0.5-unit intervals for clinical convenience. Grade 0 indicates the absence of cataract for that type. The maximum LOCS III range for nuclear cataract (N) is 6.9, which is mapped to 7 in the dataset; for cortical (C) and posterior subcapsular (P) cataract the maximum is 5.9, mapped to 6.

This study consolidates the LOCS III grades into three severity levels: None/Mild, Moderate, and Advanced. Because grade 0 (no opacity of the respective type) is included in the lowest category, this category is referred to as None/Mild. The grade boundaries were determined through consultation with an ophthalmologist and confirmed to represent clinically meaningful distinctions. N was mapped as None/Mild (0–2.0), Moderate (2.5–4.0), and Advanced (4.5–7.0); C and P share the same boundaries of None/Mild (0–2.0), Moderate (2.5–3.5), and Advanced (4.0–6.0). Because the LOCS III scale spans a wider range for N (0–7) than for C and P (0–6), the Moderate/Advanced boundary differs: grade 4.0 falls within Moderate for N but within Advanced for C and P.

**Figure 3:**
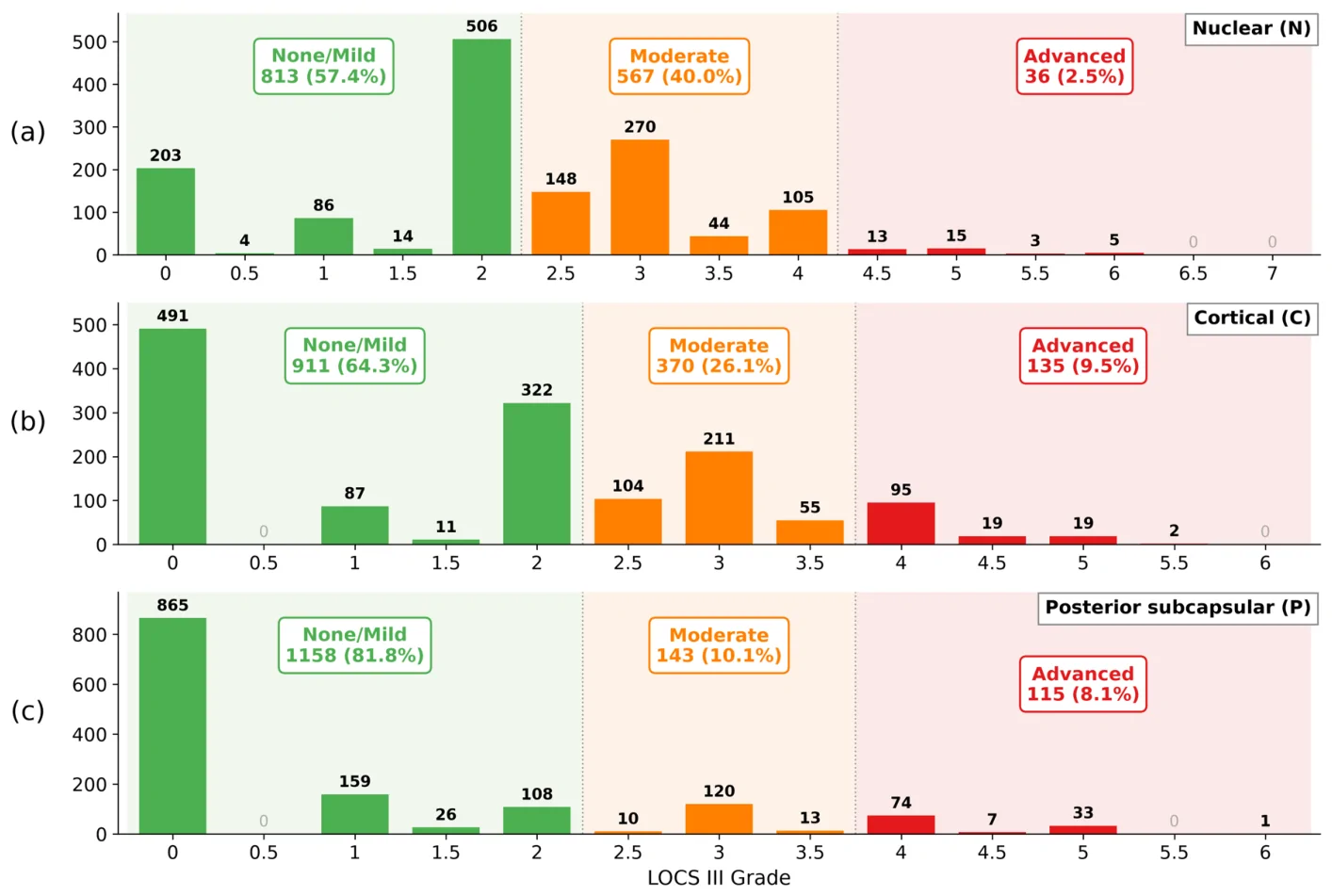
LOCS III grade distributions and three-level severity conversion: (**a**) N distribution, (**b**) C distribution, and (**c**) P distribution.

Figure 3 shows the per-grade distribution after the three-level conversion. All three types are skewed toward None/Mild, with the most pronounced imbalance in N Advanced (36 cases, 2.5%) and P Advanced (115 cases, 8.1%).

Two considerations motivate the three-level consolidation. First, distinguishing adjacent 0.5-unit grades from AS-OCT images has been a persistent challenge in automatic grading [7,8]. Second, at 0.5-unit granularity several grades contain fewer than five samples (Figure 3), making stable learning impractical.

### 3.4. Model Architecture

**Figure 4:**
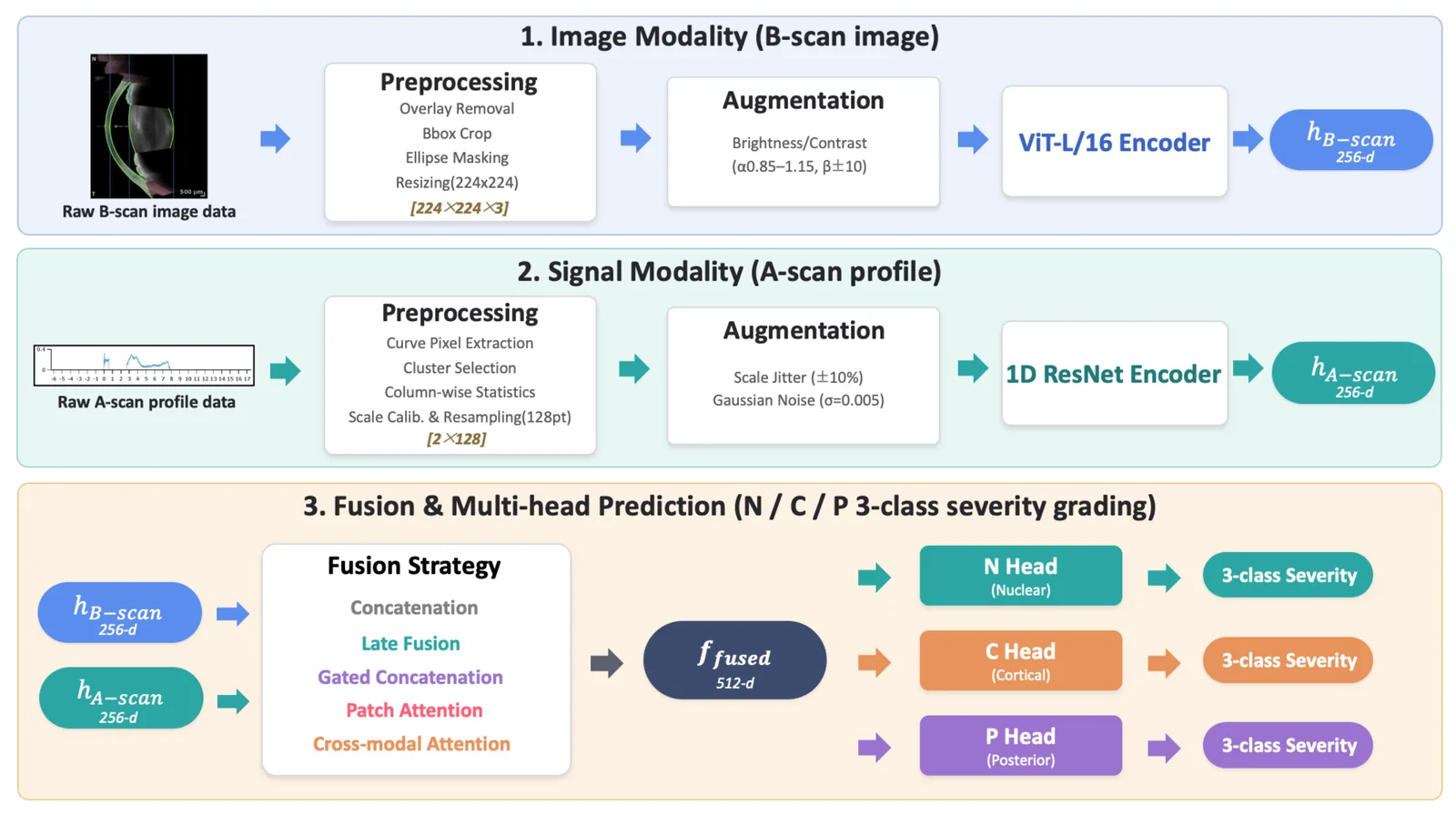
Proposed multimodal classification framework.

The proposed multimodal classification framework consists of two independent encoder pipelines that process the B-scan image and A-scan profile separately, and a multi-head structure that fuses their outputs to simultaneously predict the severity of all three components (Figure 4). The overall pipeline comprises three stages: (1) image-modality preprocessing and encoding, (2) signal-modality preprocessing and encoding, and (3) multimodal fusion and multi-head prediction.

#### 3.4.1. B-Scan Encoding Pipeline

After overlay removal and region-of-interest extraction (Section 3.2), the B-scan image undergoes data augmentation consisting of brightness and contrast jittering (α∈[0.85,1.15], β∈[−10,10]). Horizontal flipping is not applied because the horizontal axis of the B-scan image corresponds to the anterior–posterior direction of the lens; flipping would reverse the anterior and posterior lens anatomy, which is essential for identifying posterior subcapsular opacities.

For encoding, an ImageNet-pretrained Vision Transformer (ViT-Large/16) [33] is used. The 224×224 input is divided into 196 patches of size 16×16, and inter-patch relationships are learned through 24 Transformer blocks. The CLS token output (1024 dimensions) serves as the global image representation and is projected through a linear layer (Linear–ReLU–Dropout) to a 256-dimensional representation vector ($h_{\text{B-scan}}$).

#### 3.4.2. A-Scan Profile Encoding Pipeline

After two-channel resampling (Section 3.2), the A-scan profile is represented as a 2×128 tensor ($y_{\mathrm{mean}}$, $y_{\mathrm{std}}$). During training, scale jittering (±10%) and Gaussian noise (σ=0.005) are applied; the profile is not reversed to preserve its anterior-to-posterior order.

A one-dimensional residual network (1D-ResNet) [34] is designed for encoding. The stem stage applies a kernel-7 one-dimensional convolution (2 channels → 64 channels) followed by max pooling, reducing the length from 128 to 64 points. Four subsequent stages, each containing two residual blocks, progressively reduce the representation: stages 1–3 apply stride-2 convolution downsampling, increasing the channel count (64→128→256→256) while decreasing the length (64→32→16→8 points). Stage 4 applies residual blocks without downsampling to refine the representation. Global average pooling produces the final 256-dimensional representation vector ($h_{\text{A-scan}}$). Each residual block consists of two Conv1d–BatchNorm–ReLU layers with an identity shortcut. Because no large-scale pretrained resource exists for A-scan profiles, the network is trained from random initialization; the small input dimensionality makes this feasible.

#### 3.4.3. Multimodal Fusion and Multi-Head Prediction

The 256-dimensional representation vectors from the two encoders ($h_{\text{B-scan}}$, $h_{\text{A-scan}}$) are combined through a fusion module into a joint feature vector ($f_{\mathrm{fused}}$). The fused vector is shared across three independent classification heads that predict the three-level severity of N, C, and P respectively. Each head has the structure Dropout(0.3)–Linear($d_{\mathrm{in}}\to 128$)–ReLU–Dropout(0.15)–Linear(128→3), where $d_{\mathrm{in}}$ depends on the fusion strategy. This study compares five fusion strategies under identical training conditions, as described below and illustrated in Figure 4.

**Concatenation**: As the simplest feature-level strategy, the global representations from the two encoders are concatenated:

(5) $$f_{\mathrm{fused}}=\left[h_{\text{B-scan}}\;;\;h_{\text{A-scan}}\right]\in R^{512}.$$

The resulting 512-dimensional vector is fed to each of the three classification heads (512→3). This is the simplest strategy, with no additional learnable parameters in the fusion stage, and serves as the baseline.

**Late fusion**: In this decision-level strategy, each modality independently predicts N/C/P through its own classification head (256→3). A learnable per-component sigmoid weight $\alpha_{k}=\sigma \left(w_{k}\right)$ (k∈{N,C,P}) then produces a weighted average of the two sets of logits: $z_{k}=\alpha_{k}z_{k}^{\mathrm{oct}}+\left(1-\alpha_{k}\right)\;z_{k}^{\mathrm{sig}}$. Because fusion occurs only at the decision level, each modality’s discriminative capacity is preserved, and the learned $\alpha_{k}$ values allow post-hoc interpretation of per-component modality contributions.

**Gated concatenation**: In this gate-based strategy, a learnable sigmoid gate per component controls the contribution of the signal representation. The concatenated vector (512 dimensions) is passed through a nonlinear projection to produce a per-component scalar gate $g_{k}=\sigma \;\left(f_{k}\left(\left[h_{\mathrm{oct}};\;h_{\mathrm{sig}}\right]\right)\right)$. The gated signal representation $g_{k}\cdot h_{\mathrm{sig}}$ is recombined with the image representation and fed to the classification head. When $g_{k}$ approaches zero, the prediction for that component becomes effectively equivalent to the image-only condition, suppressing interference from uninformative signal content.

**Patch attention**: In this attention-based strategy, the global signal output $h_{\mathrm{sig}}\in R^{256}$ is passed through a nonlinear projection to generate 196-dimensional attention weights, which are softmax-normalized and used as a weighted sum over the 196 B-scan patch tokens to reconstruct the image representation ${\tilde{h}}_{\mathrm{oct}}=\sum_{i=1}^{196}a_{i}p_{i}$, where $a=\mathrm{softmax}(f\left(h_{\mathrm{sig}}\right))$. The reconstructed image representation (256 dimensions) is concatenated with the global signal representation to form a 512-dimensional vector for the classification heads. The signal guides the spatial focus on the image, but a single attention map is shared across N, C, and P without component-specific differentiation.

**Cross-modal attention**: This attention-based strategy uses anatomical region definitions (Section 4.3.1) to construct component-specific query tokens. Based on the fractional boundaries computed from the anterior capsule peak *a* and posterior capsule peak *p* (lens length l=p−a), segments corresponding to N ([a+0.20l,a+0.80l]), C (anterior [a,a+0.30l] and posterior [a+0.70l,p]), and P ([a+0.85l,p]) are extracted from the raw A-scan signal. Average pooling and independent nonlinear projections are applied to each segment, producing four query tokens $Q=\left[q_{N},\;q_{C_{\mathrm{ant}}},\;q_{C_{\mathrm{post}}},\;q_{P}\right]\in R^{4\times 256}$. The two cortical queries are averaged into a single representation for the C component. Each query attends to the 196 B-scan patch tokens as keys and values via multi-head cross-attention: $O_{k}=\mathrm{CrossAttn}\left(q_{k},\;P_{\mathrm{oct}}\right)$. A residual connection, LayerNorm, and FFN are applied sequentially to each component’s cross-attention output, which is then concatenated with the global signal representation to form a 512-dimensional input for the component-specific classification head. The key difference from patch attention is that anatomical region information is used explicitly, allowing each component to independently learn which spatial regions of the image are relevant to its specific signal characteristics.

### 3.5. Use of Generative AI

Claude Opus 5.5 (Anthropic) was used to translate the manuscript from Korean into English, to edit the text, and to check the bibliography against public registries; all outputs were reviewed and edited by the authors.

## 4. Experiments

This section describes the training and evaluation setup and the experimental design. The experiments serve three objectives. First, we examine whether adding the A-scan profile improves performance over the B-scan image alone. Second, the five fusion strategies described in Section 3.4.3 are compared under identical conditions to analyze how each strategy combines the two representations. Third, we verify whether the model actually exploits the A-scan profile and whether the pattern of exploitation is consistent with the geometric relationship between opacity location and the visual axis. All experiments share the same cross-validation splits and training configuration.

### 4.1. Training Strategy

**Two-phase training**: To preserve the pretrained backbone representations while preventing overfitting on the limited dataset, training is divided into two phases. In Phase 1, the ViT-L/16 backbone is frozen and only the classification heads and the signal encoder are trained, so that the randomly initialized components do not perturb the backbone representations. In Phase 2, all parameters are unfrozen, but a lower learning rate is applied to the backbone to suppress abrupt changes to the pretrained representations.

**Ordinal label smoothing**: Because cataract severity is an ordinal scale, penalizing confusion between adjacent grades and distant grades equally is inappropriate. To reflect this, Gaussian soft labels are used instead of one-hot labels. A Gaussian soft label distributes probability mass according to the distance between grades, thereby encoding ordinal information directly into the loss function while retaining the standard cross-entropy framework, which allows seamless integration with per-component classification heads and class-weighted losses. The soft label for ground-truth class *c* is defined as

(6) $$p_{k}=\frac{\exp\;\left(-\frac{{\left(k-c\right)}^{2}}{2\sigma^{2}}\right)}{\sum_{j}\exp\;\left(-\frac{{\left(j-c\right)}^{2}}{2\sigma^{2}}\right)},$$

where *k* is the class index and σ controls the spread of the distribution. Larger σ assigns more probability mass to adjacent grades. We use σ=0.5; an additional sweep over {0.3,0.5,0.7} is reported in Section 5.4.2 to justify this choice. At σ=0.5, the endpoint grades (None/Mild, Advanced) assign approximately 88% to the correct class and 12% to the adjacent class, while the middle grade (Moderate) assigns approximately 79% to the correct class and 11% to each neighbor. Classes two or more steps away receive less than 0.03%, preserving ordinal structure without excessive smoothing.

**Loss function**: The overall loss is the sum of soft cross-entropy losses for the three components:

(7) $$L=L_{N}+L_{C}+L_{P},\;L_{\mathrm{comp}}=-\sum_{k}{\tilde{w}}_{k}\;p_{k}^{\mathrm{soft}}\log{\hat{p}}_{k},$$

where $p_{k}^{\mathrm{soft}}$ is the soft label from Equation (6), ${\hat{p}}_{k}$ is the predicted probability, and ${\tilde{w}}_{k}$ is the normalized class weight. To mitigate the class imbalance visible in Figure 3, independent inverse-square-root weights $w_{k}\propto 1/\sqrt{n_{k}}$ are computed for each component. The main training settings and implementation environment are summarized in Table 2.

### 4.2. Evaluation Protocol

**Cross-validation**: Five-fold StratifiedGroupKFold cross-validation (random_state=42) is used to evaluate generalization performance. Patient-level grouping ensures that both eyes and all repeat examinations of the same patient are never split across the training and test sets, preventing data leakage. Stratification is based on the integrated severity defined as

(8) $$S_{\mathrm{int}}=\max\left(y_{N},\;y_{C},\;y_{P}\right),$$

which avoids the sparse-combination problem that arises when stratifying over all 27 possible N/C/P combinations simultaneously. The resulting fold-level distribution is 35.5% None/Mild, 46.9% Moderate, and 17.6% Advanced. Within each outer fold, the training set is further split by an inner 4-fold StratifiedGroupKFold (75% training, 25% validation), maintaining the same patient-level grouping and stratification criterion. Early stopping with patience 15 is applied based on the component-averaged Macro F1 on the validation set, and final performance is computed on the held-out outer test fold.

**Metrics**: Macro F1-score is the primary metric, chosen for its robustness to class imbalance. Overall accuracy and AUPRC (Area Under the Precision–Recall Curve), which reflects precision and recall for minority classes, are reported as secondary metrics. All metrics are computed per component (N, C, P) and averaged.

**Reporting**: All results are reported as the mean and standard deviation (mean ± SD) across the five folds.

### 4.3. Experimental Design

#### 4.3.1. Anatomical Region Definition

Prior to describing the individual experiments, this section defines the cataract-type-specific regions on the A-scan profile that are used both for constructing the cross-modal attention queries (Section 3.4.3) and for interpreting the masking experiments (Section 4.3.3).

**Figure 5:**
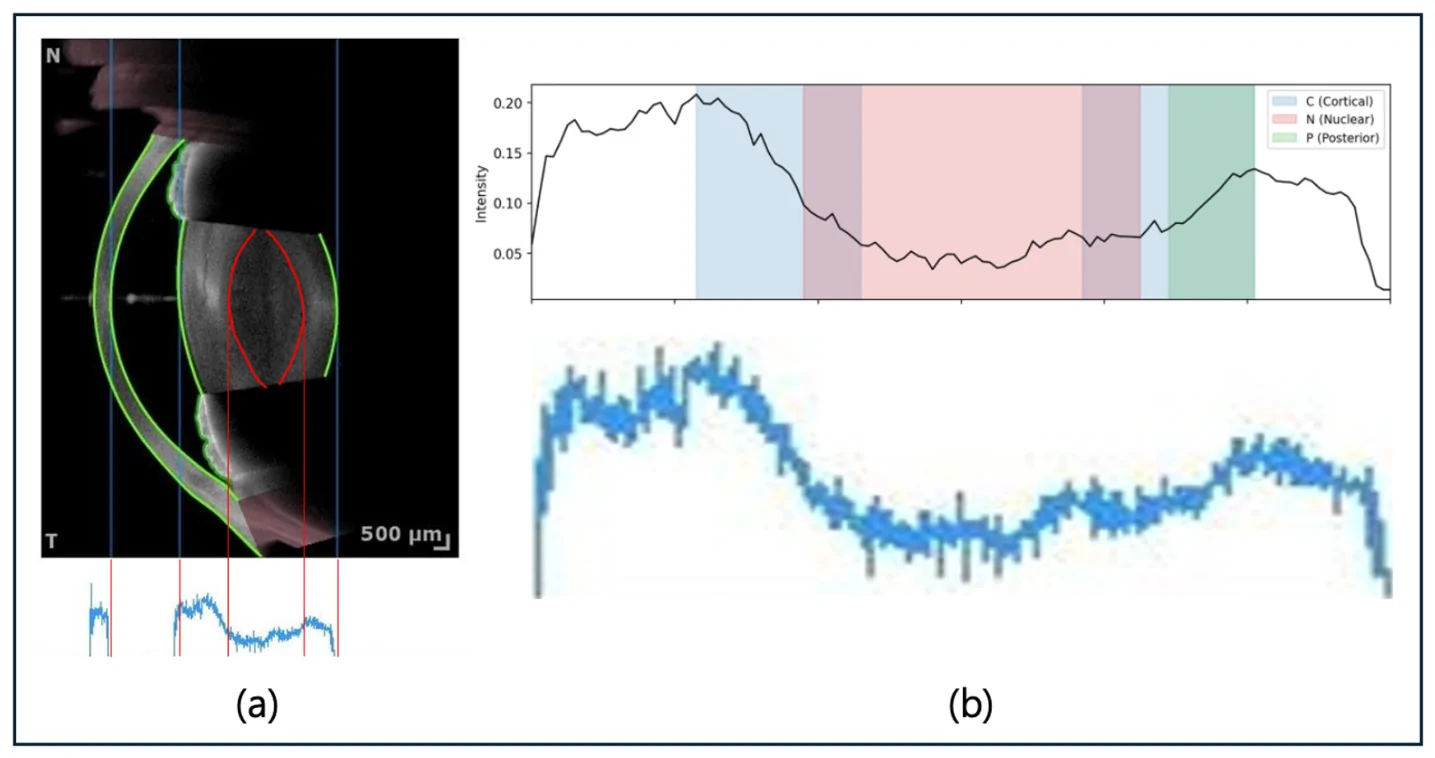
Correspondence between the B-scan image and the A-scan intensity profile. (**a**) The A-scan profile is aligned below the lens region (central green ellipse) in the AS-OCT B-scan; the nuclear region (red ellipse) lies between the two capsular peaks. (**b**) Top: anatomical regions defined from the automatically detected peaks on the A-scan profile (N: nuclear, C: cortical, P: posterior subcapsular). Bottom: A-scan profile image.

Figure 5 illustrates the B-scan image and the corresponding A-scan intensity profile for a single examination. As shown in Figure 5a, the nuclear region of the lens lies between two peaks formed by sharp increases in reflectance at the anterior and posterior capsules. Using this characteristic, the anterior and posterior capsule peaks are detected automatically, and fractional boundaries corresponding to the anatomical locations of each cataract type [4] are computed (Figure 5b, background shading).

**Automatic peak detection**: The A-scan profile is min-max normalized to [0,1], and peaks are detected using scipy.signal.find_peaks with height ≥0.3, prominence ≥0.05, and minimum spacing of 3 points. Among the detected peaks, the tallest within the anterior 30% of the profile is selected as the anterior capsule peak, and the tallest within the posterior 30% as the posterior capsule peak. For the 3 examinations in which severe cataract-induced profile distortion prevented peak detection, fixed values based on the dataset-wide mean peak positions are applied.

**Region definition:** Let *a* and *p* denote the anterior and posterior capsule peak positions, respectively, and l=p−a the lens length. The regions corresponding to each cataract type are computed as follows, with all boundaries expressed as fractions of the full profile length *L*:

- **Nuclear (N):** [a+0.20l,a+0.80l]. The central 60% of the lens length, covering the nucleus where nuclear sclerosis occurs.
- **Anterior cortex (C_ant_):** [a,a+0.30l]. From the anterior capsule peak to 30% of the lens length, corresponding to the anterior cortical region. Overlaps with the N region by 10%.
- **Posterior cortex (C_post_):** [a+0.70l,p]. From 70% of the lens length to the posterior capsule peak, corresponding to the posterior cortical region. Overlaps with the N region by 10%.
- **Posterior subcapsular (P):** [a+0.85l,p]. From 85% of the lens length to the posterior capsule peak, a narrow region reflecting the anatomical location of posterior subcapsular cataract just anterior to the posterior capsule. Overlaps with C_post_ by 15%.

Peak detection is performed automatically from the signal characteristics, whereas the fractional boundaries (20%, 30%, 70%, 85%) are set to reflect the anatomical locations of each cataract type: nuclear sclerosis in the lens center, cortical opacity near the anterior and posterior capsules, and posterior subcapsular opacity just anterior to the posterior capsule. The specific values were determined empirically through data observation. Because anatomical boundaries between cataract types are not clinically sharp, particularly in the transition zones between the cortex and nucleus and between the cortex and posterior subcapsular region where opacities of both types can coexist, 10–15% overlap between adjacent regions is deliberately introduced. This overlap accommodates boundary ambiguity and prevents information loss from rigid partitioning.

#### 4.3.2. Single-Modality Contribution

To determine whether adding the A-scan profile improves performance over the B-scan image alone, two single-modality conditions are compared. *Signal only* uses only the A-scan profile as input, passing the 1D-ResNet output to the classification heads. *OCT only* uses only the B-scan image, with the ViT-L/16 CLS token as the representation. All training conditions other than the encoder and head configuration are held constant.

#### 4.3.3. A-Scan Profile Contribution Analysis

The following two experiments directly test the central hypothesis that the A-scan profile’s contribution depends on the geometric relationship between opacity location and the visual axis.

**Region-wise profile masking**: To identify which regions of the profile the model relies on, specific segments are masked (zeroed out) at inference time on the trained concatenation model, and the resulting per-component performance change is measured. The concatenation model is chosen to exclude inter-modal interactions introduced by more complex fusion strategies, thereby isolating the contribution of individual signal regions. If the location-dependent hypothesis holds, nuclear classification should be selectively sensitive to masking the central region, posterior subcapsular to the posterior region, and cortical should show minimal change regardless of which region is masked. Rather than using the type-specific regions defined in Section 4.3.1, the 128-point profile is divided into 10 equal segments (each approximately 12.8 points), and each segment is masked independently. This uniform partition enables continuous observation of position-dependent sensitivity across the entire profile.

**Extraction pathway comparison**: The Anterion device provides a single A-scan intensity profile extracted along the visual axis. Because an AS-OCT B-scan comprises multiple axial A-scan lines, it is possible that a suitably chosen line from the B-scan could substitute for the device-provided profile. If equivalent information can be obtained from the B-scan alone, the rationale for treating the A-scan profile as a separate modality would be weakened.

To test this, three alternative line profiles are extracted from the preprocessed B-scan crop (Section 3.2.1) before resizing, resampled to 128 points via linear interpolation identically to the A-scan preprocessing:

- **A-scan profile:** the device-provided profile along the visual axis (reference).
- **Best-corr:** the B-scan row with the highest Pearson correlation to the A-scan profile.
- **Widest:** the row at the widest horizontal extent of the lens.
- **Center:** the row at the geometric center of the lens region.

**Figure 6:**
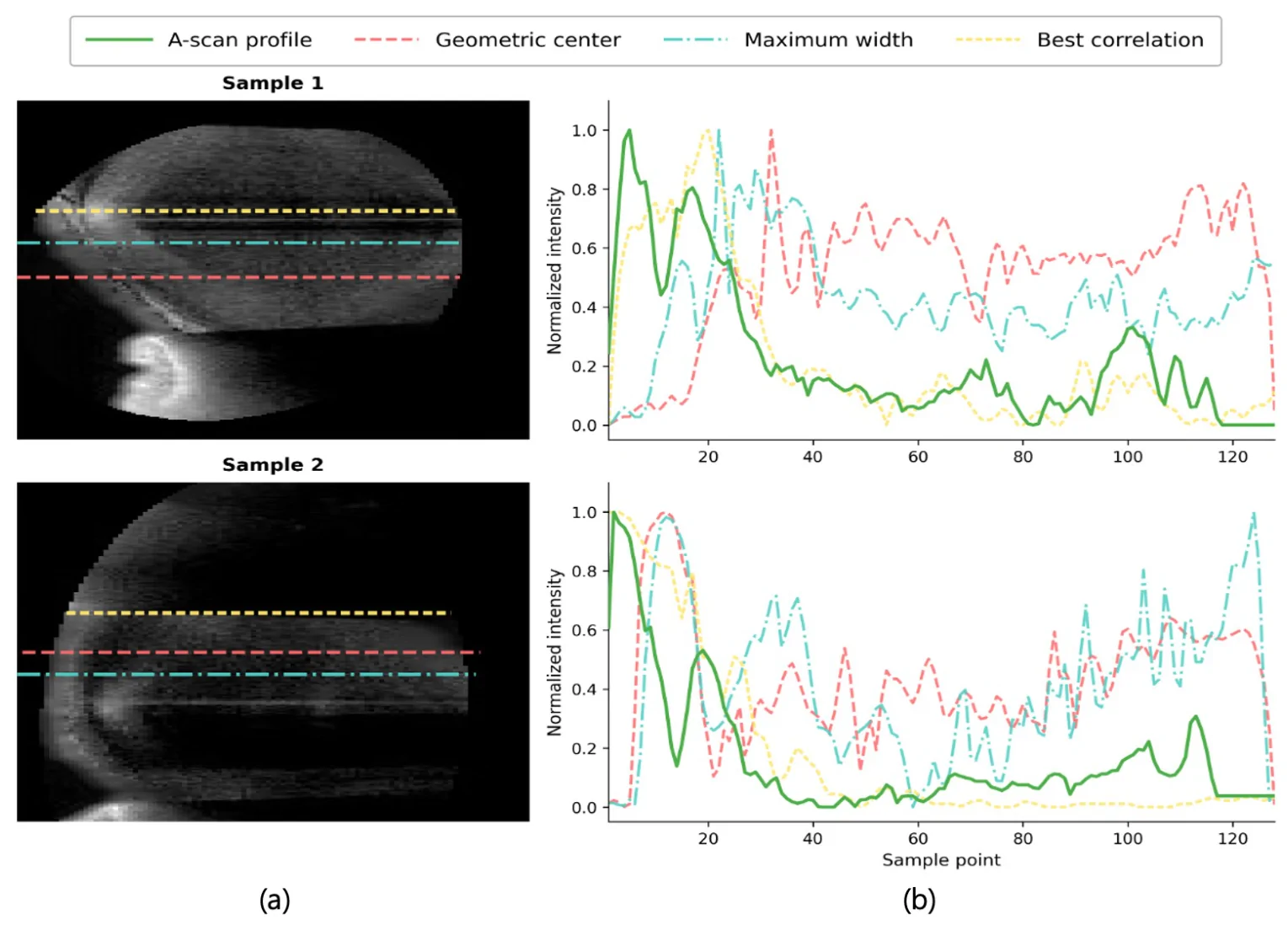
(**a**) Three line extraction positions on the cropped B-scan image. (**b**) Comparison with the A-scan profile.

Figure 6 compares the three B-scan-derived profiles against the A-scan profile for two representative samples, showing qualitative differences in peak amplitude and decay patterns. Each alternative profile is substituted for the device-provided A-scan and evaluated under the same training and evaluation protocol. Best-corr selects the most similar row post-hoc using the device-provided A-scan as the reference, so it cannot be used at inference time; it serves as an upper bound on the performance achievable from B-scan-derived profiles. If this upper bound falls below the device-provided A-scan, it indicates that the B-scan alone cannot reproduce the information obtained through the device’s visual-axis alignment and signal processing, supporting the use of the A-scan profile as an independent input.

#### 4.3.4. Ablation Studies

**Backbone comparison**: To assess the effect of the pretraining strategy and architecture of the image encoder, the default ImageNet-pretrained ViT-L/16 is replaced with RETFound [35], a retinal OCT-specialized foundation model, and with ResNet-34, a CNN baseline. The RETFound comparison tests whether domain-specific pretraining on retinal OCT transfers effectively to AS-OCT, while the ResNet comparison quantifies the Transformer-CNN gap.

**Soft-label σ sweep**: To justify the choice of σ=0.5 for the Gaussian soft label, performance is compared across σ∈{0.3,0.5,0.7}.

**Figure 7:**
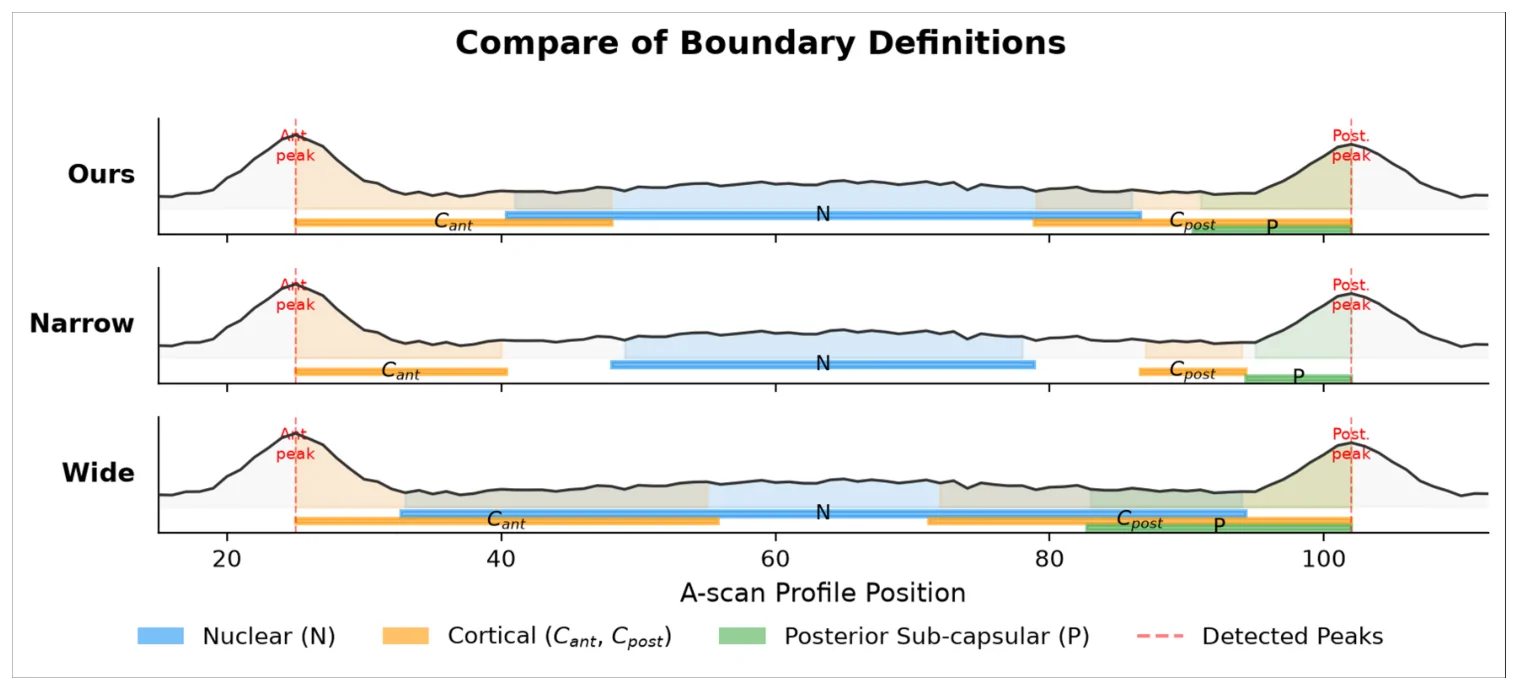
Three region definitions compared: Ours, Narrow, and Wide.

**Region sensitivity comparison**: To analyze the effect of the anatomical region boundaries defined in Section 4.3.1 on the performance of cross-modal attention, three region settings are compared (Figure 7). The proposed setting (*Ours*: N 20–80%, C_1_ 0–30%, C_2_ 70–100%, P 85–100%) includes deliberate 10–15% overlap to reflect the clinically ambiguous boundaries between adjacent structures. The *Narrow* setting removes all overlap and contracts each region (N 30–70%, C_1_ 0–20%, C_2_ 80–90%, P 90–100%), enforcing complete separation. The *Wide* setting expands each region and increases overlap (N 10–90%, C_1_ 0–40%, C_2_ 60–100%, P 75–100%). This comparison tests whether the proposed level of overlap contributes to the cross-modal attention performance.

## 5. Results

### 5.1. Modality Contribution and Fusion Strategies

Table 3 compares the 5-fold cross-validation performance of two single-modality conditions and five fusion strategies. Signal only recorded an average F1 of 0.463 and Acc of 0.667, while OCT only achieved an average F1 of 0.490 and Acc of 0.693, outperforming Signal only in average F1, Acc, and AUPRC. All five fusion strategies exceeded both single-modality conditions in average F1 (0.514–0.531), Acc (0.699–0.708), and AUPRC (0.547–0.564). Concatenation and Late fusion achieved the highest average F1 (0.531), Patch attention the highest average AUPRC (0.564), and Cross-modal attention the highest average Acc (0.708); the differences in average F1 among the fusion strategies (at most 0.017) were smaller than the inter-fold standard deviations (0.023–0.035). Across components, the largest improvement was observed for Posterior, where F1 increased from 0.469 (OCT only) to 0.539–0.580 under all fusion strategies, and Nuclear also exceeded OCT only (0.504) under four of the five strategies (0.513–0.546). In contrast, Cortical remained at a level similar to OCT only (0.497; fusion strategies 0.461–0.513).

**Figure 8:**
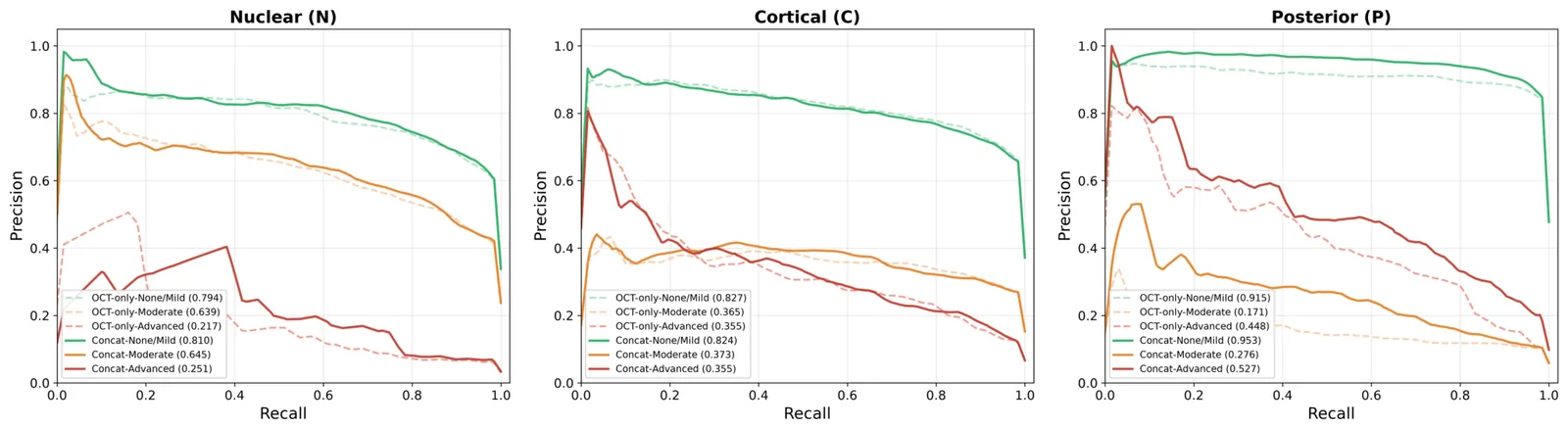
Per-component, per-grade precision–recall curves for OCT only and Concatenation (5-fold cross-validation average; per-grade AUPRC in parentheses).

Table 4 compares the per-grade performance of OCT only and Concatenation, and Figure 8 shows the corresponding precision–recall curves. For the P component, Concatenation improved F1 at all three grades, with the largest gain at the Moderate grade (F1 from 0.183 to 0.336, Recall from 0.228 to 0.424, AUPRC from 0.171 to 0.276); Advanced-grade F1 and AUPRC increased from 0.342 to 0.454 and from 0.448 to 0.527, respectively, and None/Mild-grade AUPRC increased from 0.915 to 0.953. For the N component, Concatenation improved Moderate-grade F1 from 0.574 to 0.620 and Recall from 0.527 to 0.621; at the Advanced grade, F1 increased from 0.177 to 0.229, but with large standard deviations reflecting the small number of cases. For the C component, Moderate-grade F1 increased from 0.395 to 0.436, whereas Advanced-grade F1 decreased from 0.328 to 0.296, and the PR curves of the two conditions largely overlapped at the Moderate and Advanced grades.

### 5.2. Misclassification Patterns

**Figure 9:**
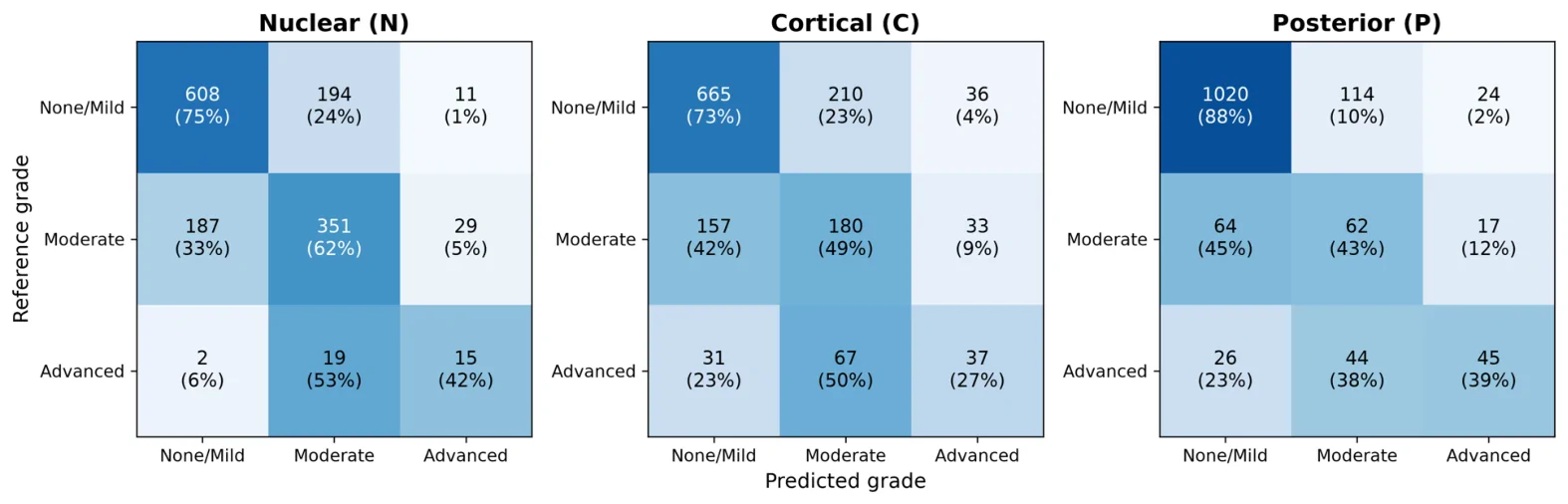
Per-component confusion matrices of the concatenation model, pooled over the five test folds (1416 examinations). Each cell shows the number of examinations and the percentage of the reference grade.

Figure 9 shows the per-component confusion matrices of the concatenation model, obtained by pooling the out-of-fold predictions of all five test folds so that each of the 1416 examinations appears once. Most misclassifications occurred between adjacent grades (97.1%, 87.5%, and 82.7% of all errors for N, C, and P, respectively), and two-step errors between None/Mild and Advanced accounted for 0.9%, 4.7%, and 3.5% of all predictions. Of these two-step errors, Advanced examinations predicted as None/Mild numbered 2, 31, and 26, and None/Mild examinations predicted as Advanced numbered 11, 36, and 24, for N, C, and P, respectively.

### 5.3. A-Scan Profile Contribution Analysis

#### 5.3.1. Region-Wise Profile Masking

**Figure 10:**
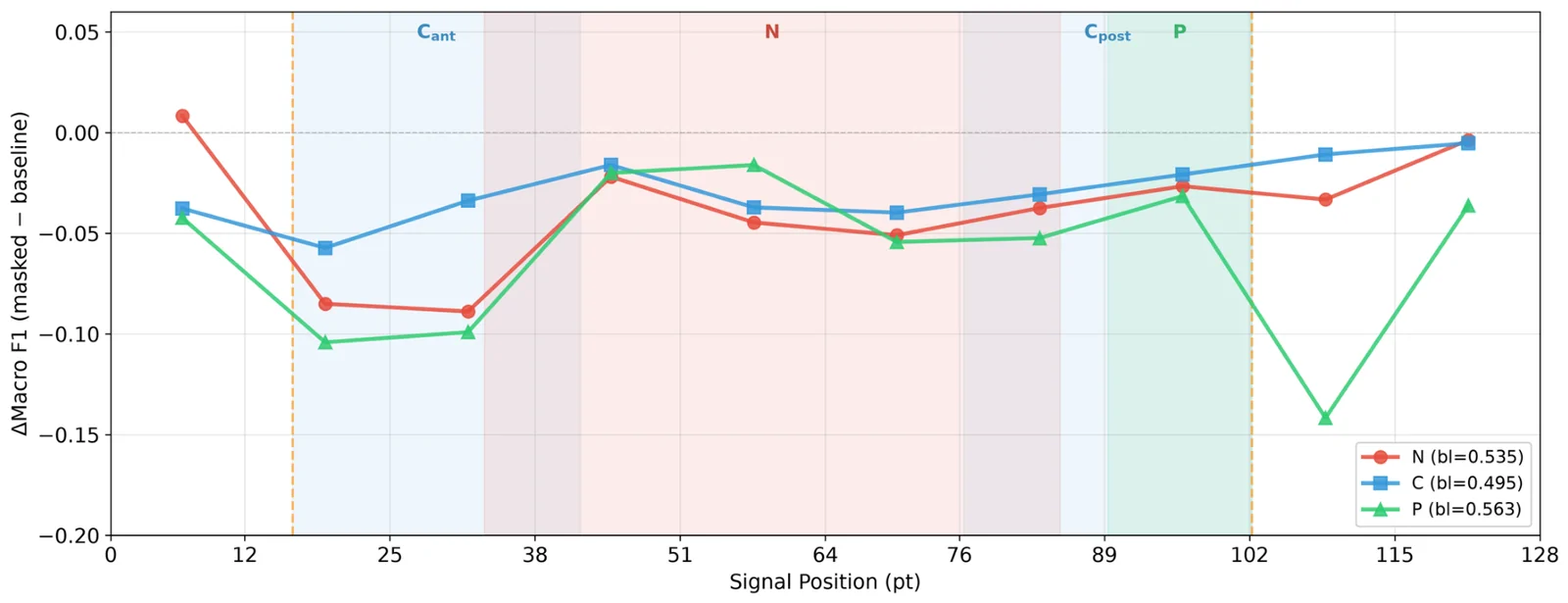
Region-wise masking results. The horizontal axis represents the 128-point A-scan profile length; the vertical axis shows the ΔF1 relative to the baseline concatenation model. C_ant_: anterior cortex, N: nuclear, C_post_: posterior cortex, P: posterior subcapsular.

Table 5 reports the F1 and ΔF1 for each of the 10 masked segments, and Figure 10 plots the ΔF1 profile for each component. ΔF1 is defined as the masked F1 minus the baseline (concatenation) F1; larger negative values indicate greater contribution of that segment to classification.

The P component showed the largest single drop among all components when the segment containing the posterior capsule was masked (102–115 pt, ΔF1 =−0.142; mean posterior capsule peak at 102.2 pt) (Table 5, Figure 10). The N component showed the largest drops when the anterior-to-early-central segments (12–38 pt) were masked (ΔF1 =−0.085 and −0.089), followed by the central segments (51–89 pt; ΔF1 =−0.037 to −0.051), whereas masking either end of the profile (0–12 and 115–128 pt) had little effect (+0.008 and −0.004). The C component showed smaller decreases across segments (ΔF1 =−0.005 to −0.057), with the largest at the anterior cortex segment (12–25 pt).

#### 5.3.2. Extraction Pathway Comparison

**Figure 11:**
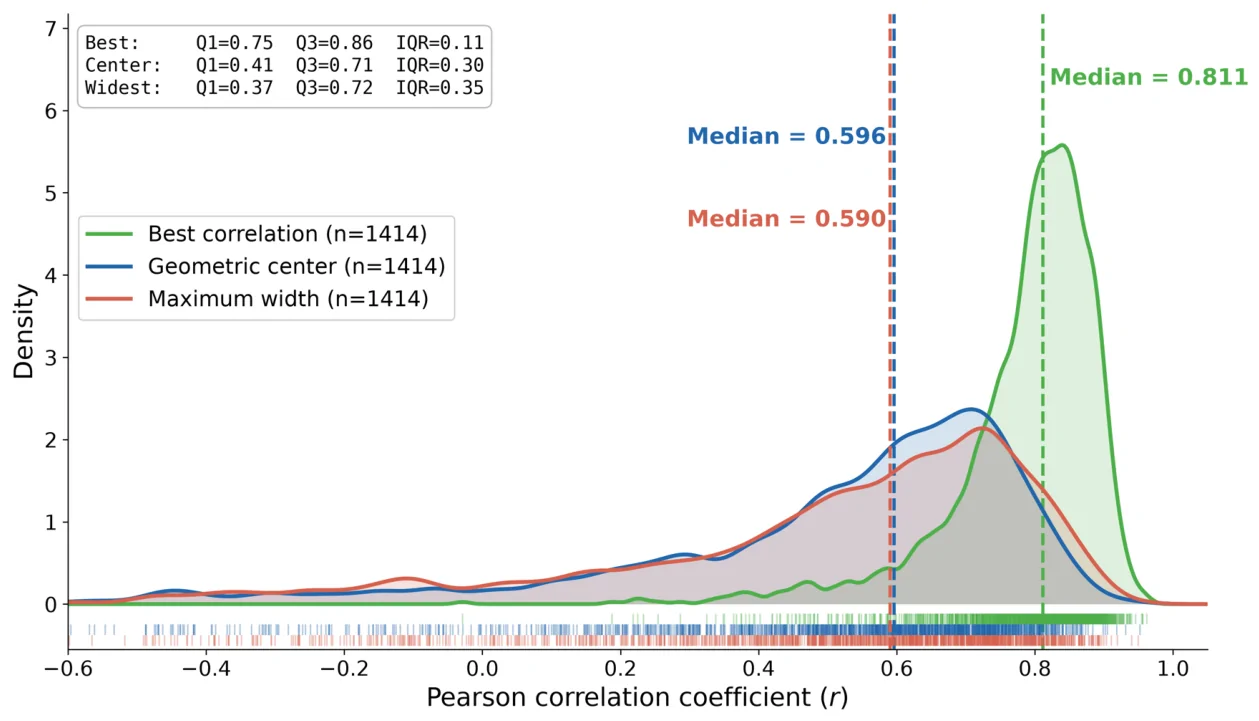
Pearson correlation distributions between the device-provided A-scan profile and line profiles extracted from the B-scan image, computed for three row-selection criteria: geometric center, maximum width, and best correlation.

Figure 11 shows the distribution of Pearson correlation coefficients between the device-provided A-scan profile and three B-scan-derived line profiles across 1414 examinations (two examinations with failed elliptical lens masking were excluded). Geometric center (median r=0.596) and maximum width (median r=0.590) showed moderate correlation, and even the post-hoc best-corr condition achieved only a median of r=0.811, indicating that the device-provided profile cannot be fully reproduced from a single B-scan row.

Table 6 compares the classification performance of the concatenation model when the device-provided A-scan profile was replaced with each of the three B-scan-derived line profiles. The device-provided A-scan profile achieved the highest average F1 (0.531), Acc (0.702), and AUPRC (0.557), exceeding best-corr (F1 0.489), widest (0.482), and center (0.478). Per component, the device-provided A-scan profile achieved the highest F1 for all three components (N: 0.535 vs. next-best 0.517; C: 0.495 vs. 0.470; P: 0.563 vs. 0.479), with the largest margin for P.

### 5.4. Ablation Studies

#### 5.4.1. Backbone Comparison

Table 7 compares classification performance across backbone models. Under the OCT-only condition, ViT-L/16 (ImageNet) achieved an average F1 of 0.490, compared with 0.486 for RETFound and 0.434 for ResNet-34. Under concatenation, ViT-L/16 (ImageNet) achieved the highest average F1 (0.531), followed by RETFound (0.504) and ResNet-34 (0.498). Adding the A-scan profile improved average F1 for all three backbones (ResNet-34: +0.064; ViT-L/16 (ImageNet): +0.041; RETFound: +0.018).

#### 5.4.2. Soft-Label σ Sweep

As shown in Table 8, σ≥0.7 caused excessive label smoothing and a sharp drop in classification performance (average F1 0.400). σ=0.3 and σ=0.5 achieved similar average F1 scores (0.540 and 0.531), with a difference smaller than the inter-fold standard deviations of each component (0.041–0.066). Because σ=0.3 assigns more than 99% of the probability to the correct class and thus approximates one-hot labels, σ=0.5 was retained as the setting that preserves ordinal smoothing between adjacent grades.

#### 5.4.3. Region Sensitivity

Table 9 compares cross-modal attention performance across the three region settings. The three settings achieved similar average F1 (Ours 0.525 ± 0.035, Narrow 0.529 ± 0.024, Wide 0.539 ± 0.032); the largest difference (0.014) was smaller than the inter-fold standard deviations, indicating that cross-modal attention performance was not sensitive to the exact definition of the region boundaries.

## 6. Discussion

This study designed a multimodal approach and compared five fusion strategies based on the hypothesis that the B-scan image and A-scan profile provide type-dependent contributions to cataract severity classification. Specifically, nuclear cataract (N) and posterior subcapsular cataract (P), whose opacities are traversed by the visual axis, were expected to benefit from the axial intensity information in the A-scan profile, while cortical cataract (C), which extends asymmetrically away from the visual axis, was expected to rely primarily on the B-scan image. This section examines whether the experimental results support this hypothesis and discusses observations that deviate from the prediction.

**Modality contribution and fusion strategies**: Adding the A-scan profile produced performance gains for N (ΔF1 =+0.030) and P (ΔF1 =+0.094), but no meaningful improvement for C (ΔF1 =−0.002), in agreement with the hypothesis. In the fusion strategy comparison, all five fusion strategies outperformed both single-modality conditions, and their average F1 differed by at most 0.017, less than the inter-fold standard deviations. The gain for P was observed under every fusion strategy (F1 from 0.469 to 0.539–0.580), indicating that the improvement derives from the information contained in the A-scan profile itself rather than from a particular fusion architecture. Accordingly, the simplest strategy, concatenation, was sufficient to exploit this complementary information, and the attention-based strategies did not provide a consistent additional benefit in this dataset.

**Performance level and misclassification patterns**: The absolute performance of the models remained modest (average macro F1 of approximately 0.53), and the present results should be interpreted as evidence of the added value of the A-scan profile rather than as an indication that the system is ready for routine clinical grading. The confusion matrices (Figure 9) showed that most misclassifications occurred between adjacent grades (82.7–97.1% of all errors), whereas two-step errors between None/Mild and Advanced accounted for 0.9–4.7% of all predictions. Adjacent-grade boundaries are also where the reference grading itself is least reproducible: in the inter-observer analysis, most disagreements in N occurred between adjacent LOCS III scores (2.0 vs. 2.5; Table 1). Performance also differed considerably across grades, and the less frequent categories remained difficult to classify; with the concatenation model, F1 was 0.229 for N Advanced (36 cases), 0.336 for P Moderate (143 cases), and 0.296 for C Advanced (135 cases), compared with 0.899 for P None/Mild. Clinical interpretation should therefore be based on per-grade performance rather than on the average F1 alone. Notably, the contribution of the A-scan profile was most evident in the minority grades of P: Moderate-grade F1 increased from 0.183 to 0.336, Advanced-grade F1 from 0.342 to 0.454, and the number of P Advanced examinations predicted as None/Mild decreased from 34 to 26.

**Region-wise profile masking**: The region-wise masking experiment confirmed the component-specific dependence on the A-scan profile at the segment level. For P, the largest drop among all components occurred when the segment containing the posterior capsule was masked (102–115 pt, ΔF1 =−0.142). Although this segment lies just posterior to the mean posterior capsule peak (102.2 pt), the posterior capsule peak in eyes with Moderate or Advanced P was located further posteriorly (median 108 and 111 pt vs. 102 pt in None/Mild), so that this segment covered the posterior subcapsular region of the affected eyes. This directly supports the hypothesis that the posterior subcapsular signal along the visual axis contributes to P classification. For N, masking the anterior-to-early-central and central segments reduced performance, whereas masking either end of the profile had little effect, consistent with the central location of nuclear opacity. For C, the decreases were smaller (ΔF1 =−0.005 to −0.057) and were not concentrated in a specific region, consistent with the peripheral and spatially scattered distribution of cortical opacities, which are not adequately captured by a one-dimensional axial profile, and supporting the view that C classification relies primarily on B-scan image information.

Masking the anterior segments (12–38 pt), where the anterior capsule peak is located, also produced large drops for N (the largest for this component) and for P (ΔF1 =−0.104 and −0.099). Because the A-scan profile is provided in arbitrary units without absolute calibration, per-scan gain variations exist; under this condition, the model likely interprets the profile through intensity relative to the anterior capsule peak rather than through absolute intensity values. The anterior peak may thus serve as an internal reference, and removing it would make the remaining signal harder to interpret. This suggests that the model relies on the anterior peak as a reference for relative intensity, not only on local information from the lesion site itself.

**Extraction pathway comparison**: The extraction pathway comparison examined whether the B-scan image alone can substitute for the device-provided A-scan profile. Even under the best-corr condition, which post-hoc selects the B-scan row most similar to the A-scan profile, the median Pearson correlation was 0.811, and classification performance (average F1 0.489) fell below that of the device-provided profile (0.531). For all three components, the device-provided profile outperformed every B-scan-derived line, with the largest margin for P (F1 0.563 vs. 0.479), whose opacity is the most narrowly localized along the visual axis. This indicates that the device-provided A-scan profile carries information that cannot be recovered from a single row of the B-scan image in the report, supporting its treatment as a separate modality.

**Backbone comparison**: The backbone comparison showed that ImageNet-pretrained ViT-L/16 achieved the highest average F1 under both conditions, although its advantage over RETFound under the OCT-only condition was small (0.490 vs. 0.486). RETFound, a foundation model pretrained on retinal OCT, did not outperform the ImageNet-pretrained model on AS-OCT B-scans, which differ from retinal OCT in both the imaged anatomy and the image characteristics; the benefit of domain-specific pretraining was therefore limited in this setting. ResNet-34 scored below both ViT-based models, yet the improvement from adding the A-scan profile was larger for ResNet-34 (ΔF1 =+0.064) than for ViT-L/16 (ImageNet) (+0.041) or RETFound (+0.018), suggesting that the complementary contribution of the A-scan profile may increase when the image encoder’s representational capacity is more limited. Notably, adding the A-scan profile improved average F1 with every backbone, indicating that its contribution is not specific to a particular image encoder.

**Region sensitivity and soft-label σ**: The three region settings yielded similar average F1 (0.525–0.539), with differences smaller than the inter-fold standard deviations. Because the region queries are defined as fractional positions between the detected capsule peaks, this result suggests that cross-modal attention is robust to the exact choice of region boundaries and that the anatomical prior does not need to be precisely tuned. Regarding the choice of σ=0.5 for the Gaussian soft label, σ=0.3 yielded a similar average F1 (0.540 vs. 0.531), but it assigns more than 99% of the probability to the correct class and is therefore nearly equivalent to one-hot labels. σ=0.5 was retained because it preserves ordinal smoothing between adjacent grades, whereas σ=0.7 over-smoothed the labels and degraded performance.

**Limitations and future directions**: This study has several limitations. First, the data were collected from a single institution using a single device and report format, and no external validation was performed; the applicability of the approach to other institutions and to multi-center settings therefore remains to be demonstrated. Because the A-scan profile format is specific to the Anterion report, direct generalization to other AS-OCT devices requires further validation. Second, the three-level conversion of LOCS III grades cannot capture fine-grained inter-grade differences, and the scarcity of certain classes (N Advanced: 36 cases; P Moderate: 143 cases) limits performance stability at those levels; dense cataracts were also more often excluded because of failed lens region extraction, which may have further reduced the number of Advanced cases. Third, each examination in the full dataset was graded by only one of the two ophthalmologists. Although inter-observer agreement on an independently re-graded subset was excellent (weighted κ 0.84–0.97), residual label noise cannot be excluded, particularly near grade boundaries; most disagreements in N occurred between adjacent LOCS III scores (2.0 vs. 2.5). In addition, because slit-lamp grading and Anterion imaging were separate examinations, lens opacity may have progressed between the two in the small number of cases with long intervals (9 examinations with an interval exceeding 90 days; maximum, 364 days). Fourth, the line-profile comparison with the B-scan was limited to single-row extraction; combining multiple rows or examining the relationship with the full spatial information used by the two-dimensional encoder remains to be investigated. Fifth, direct quantitative comparison with prior studies [7,15,22] was not feasible because the datasets, devices, grade systems, and target scopes differ; the performance figures reported here should therefore be interpreted in the context of the within-study comparisons rather than as benchmarks against existing methods. Sixth, the A-scan profile is normalized by a proprietary algorithm of the device and reported in arbitrary units; although it contains discriminative information for severity classification, it should not be regarded as a direct quantitative measurement of lens optical density.

Within these limitations, the present results indicate that the A-scan profile provides a useful incremental contribution to cataract severity classification in this dataset, particularly for N and P. Future work should include multi-center external validation with reference grades from multiple graders and the use of two-dimensional densitometry maps or en-face images to address the spatial limitation of the one-dimensional profile, particularly for improving C classification.

## 7. Conclusions

This study automatically extracted B-scan images and A-scan profiles from Anterion AS-OCT examination reports, encoded them with a pretrained Vision Transformer and a one-dimensional residual network, and compared five fusion strategies for simultaneously classifying the severity of N, C, and P. Through 5-fold cross-validation on 1416 examinations from 691 patients, three principal findings were obtained.

First, the contribution of the A-scan profile was type-dependent: performance improvement was observed for N and P, whose opacities lie along the visual axis, but not for C. Second, this improvement was observed under all five fusion strategies, which performed comparably, and region-wise masking showed that P classification depended most on the segment containing the posterior capsule, consistent with the anatomical location of posterior subcapsular opacity. Third, line profiles extracted from the B-scan image could not replace the device-provided A-scan profile, indicating that the A-scan profile provides information not recoverable from the cross-sectional image through the device’s visual-axis alignment and signal processing.

These findings indicate that the Anterion A-scan profile, which has been underutilized in prior work, provides a useful incremental contribution to cataract severity classification in this dataset, particularly for N and P. Multi-center external validation with reference grades from multiple graders and the use of two-dimensional densitometry maps or en-face images are needed to confirm these findings and to further improve classification, particularly for cortical cataract.

## Figures and Tables

**Table 1:** Inter-observer agreement between the original grades and an independent second grading (271 visits, 163 patients). *n*, number of visits in which both graders recorded the respective type; ICC, two-way random effects, absolute agreement, single rater; weighted κ, quadratic weights on the three-level grades.

Type	*n*	Exact LOCS III	Within ±0.5	ICC	Three-Level Agreement	Weighted κ
N (Nuclear)	253	87.7%	97.2%	0.92	92.5%	0.84
C (Cortical)	222	88.7%	99.5%	0.98	92.8%	0.92
P (Posterior)	132	90.2%	99.2%	0.99	96.2%	0.97

**Table 2:** Training configuration and implementation environment.

Parameter	Value
Image input	224×224
Signal input	2-channel ($y_{\mathrm{mean}}$ + $y_{\mathrm{std}}$), 128-point
Backbone	ImageNet ViT-L/16 (timm)
Batch size	16
Optimizer	AdamW (weight decay 0.01)
Phase 1 learning rate	$1\times 10^{-3}$
Phase 1 epochs	10
Phase 2 backbone LR	$1\times 10^{-5}$
Phase 2 head/signal encoder LR	$1\times 10^{-4}$
Phase 2 max epochs	200
LR scheduler	Cosine annealing with 10-epoch warmup
Early stopping patience	15 (validation average Macro F1)
Dropout	0.3 (head), 0.15 (final layer)
Soft label σ	0.5
Cross-validation	5-fold StratifiedGroupKFold (seed=42)
Random seed	42 (torch + numpy)
GPU	NVIDIA RTX PRO 6000 (96 GB VRAM)
Framework	PyTorch 2.11.0, CUDA 12.8

**Table 3:** Performance comparison across modality conditions and fusion strategies (5-fold cross-validation, mean ± SD). All models were trained without horizontal flipping on the final dataset of 1416 examinations. * Reference model used in the subsequent analyses. Bold indicates the best value in each column.

Method	N (Nuclear)			C (Cortical)			P (Posterior)			Average		
	F1	Acc	AUPRC	F1	Acc	AUPRC	F1	Acc	AUPRC	F1	Acc	AUPRC
*Single modality*												
Signal only	0.488 ±0.041	0.650 ±0.039	0.551 ±0.059	0.411 ±0.032	0.571 ±0.038	0.421 ±0.042	0.490 ±0.055	0.782 ±0.035	0.510 ±0.020	0.463 ±0.028	0.667 ±0.017	0.494 ±0.034
OCT only	0.504 ±0.069	0.682 ±0.025	0.550 ±0.060	0.497 ±0.029	0.633 ±0.020	0.516 ±0.031	0.469 ±0.051	0.765 ±0.023	0.511 ±0.031	0.490 ±0.033	0.693 ±0.009	0.526 ±0.023
*Multimodal fusion*												
Concatenation *	0.535 ±0.057	0.688 ±0.034	0.569 ±0.073	0.495 ±0.058	0.623 ±0.049	0.517 ±0.052	0.563 ±0.056	0.796 ±0.040	0.585 ±0.036	**0.531** ±0.034	0.702 ±0.031	0.557 ±0.022
Late fusion	0.513 ±0.057	0.681 ±0.032	0.562 ±0.087	**0.513** ±0.014	0.638 ±0.026	**0.533** ±0.045	0.567 ±0.070	0.790 ±0.042	**0.593** ±0.053	**0.531** ±0.033	0.703 ±0.024	0.563 ±0.011
Gated concatenation	0.502 ±0.089	0.667 ±0.033	0.561 ±0.091	0.461 ±0.042	0.622 ±0.028	0.494 ±0.046	**0.580** ±0.042	**0.812** ±0.039	0.585 ±0.022	0.514 ±0.023	0.700 ±0.013	0.547 ±0.016
Patch attention	**0.546** ±0.040	**0.697** ±0.033	**0.591** ±0.082	0.489 ±0.044	0.613 ±0.012	0.516 ±0.039	0.539 ±0.051	0.788 ±0.043	0.585 ±0.073	0.525 ±0.026	0.699 ±0.020	**0.564** ±0.030
Cross-modal attention	0.535 ±0.070	0.691 ±0.039	0.576 ±0.073	0.489 ±0.032	**0.642** ±0.039	0.520 ±0.035	0.551 ±0.043	0.792 ±0.035	0.554 ±0.043	0.525 ±0.035	**0.708** ±0.033	0.550 ±0.022

**Table 4:** Per-grade performance comparison between OCT only and Concatenation (5-fold cross-validation, mean ± SD).

Component	Grade	OCT Only			Concatenation		
		F1	Recall	AUPRC	F1	Recall	AUPRC
N (Nuclear)	None/Mild	0.762±0.027	0.806±0.045	0.794±0.037	0.755±0.027	0.748±0.031	0.810±0.043
	Moderate	0.574±0.020	0.527±0.043	0.639±0.046	0.620±0.046	0.621±0.066	0.645±0.043
	Advanced	0.177±0.206	0.186±0.194	0.217±0.179	0.229±0.196	0.303±0.212	0.251±0.201
C (Cortical)	None/Mild	0.767±0.033	0.767±0.069	0.827±0.052	0.752±0.039	0.734±0.091	0.824±0.054
	Moderate	0.395±0.073	0.417±0.110	0.365±0.042	0.436±0.045	0.490±0.067	0.373±0.057
	Advanced	0.328±0.104	0.317±0.123	0.355±0.097	0.296±0.107	0.272±0.118	0.355±0.123
P (Posterior)	None/Mild	0.882±0.016	0.879±0.036	0.915±0.034	0.899±0.017	0.881±0.024	0.953±0.016
	Moderate	0.183±0.088	0.228±0.104	0.171±0.041	0.336±0.095	0.424±0.096	0.276±0.066
	Advanced	0.342±0.114	0.266±0.117	0.448±0.052	0.454±0.137	0.418±0.189	0.527±0.105

**Table 5:** Region-wise profile masking results (concatenation model, all samples, 5-fold cross-validation, mean ± SD). Bold indicates the largest decrease in F1 for each component.

Segment (pt)	Nuclear (N)		Cortical (C)		Posterior (P)	
	F1	ΔF1	F1	ΔF1	F1	ΔF1
Baseline	0.535±0.057	—	0.495±0.058	—	0.563±0.056	—
0–12	0.543±0.064	+0.008	0.457±0.036	−0.038	0.520±0.039	−0.042
12–25	0.449±0.102	−0.085	0.438±0.067	−0.057	0.459±0.075	−0.104
25–38	0.446±0.075	−0.089	0.461±0.060	−0.034	0.464±0.032	−0.099
38–51	0.513±0.056	−0.022	0.479±0.040	−0.016	0.543±0.033	−0.020
51–64	0.490±0.075	−0.045	0.458±0.046	−0.037	0.547±0.018	−0.016
64–76	0.483±0.024	−0.051	0.455±0.045	−0.040	0.508±0.026	−0.054
76–89	0.497±0.060	−0.037	0.464±0.050	−0.031	0.510±0.046	−0.052
89–102	0.508±0.060	−0.027	0.474±0.052	−0.021	0.531±0.032	−0.032
102–115	0.501±0.063	−0.033	0.484±0.055	−0.011	0.421±0.023	−0.142
115–128	0.531±0.063	−0.004	0.490±0.054	−0.005	0.527±0.053	−0.036

**Table 6:** Performance comparison across extraction pathways (concatenation fusion, 5-fold cross-validation, mean ± SD). Bold indicates the best value in each column.

Experiment	N (Nuclear)			C (Cortical)			P (Posterior)			Average		
	F1	Acc	AUPRC	F1	Acc	AUPRC	F1	Acc	AUPRC	F1	Acc	AUPRC
concat (A-scan profile)	**0.535** ±0.057	**0.688** ±0.034	0.569 ±0.073	**0.495** ±0.058	0.623 ±0.049	0.517 ±0.052	**0.563** ±0.056	**0.796** ±0.040	**0.585** ±0.036	**0.531** ±0.034	**0.702** ±0.031	**0.557** ±0.022
concat_row_best_corr	0.517 ±0.075	0.667 ±0.039	**0.572** ±0.100	0.470 ±0.061	**0.624** ±0.026	0.508 ±0.047	0.479 ±0.043	0.741 ±0.024	0.525 ±0.034	0.489 ±0.017	0.677 ±0.016	0.535 ±0.022
concat_row_widest	0.517 ±0.084	0.671 ±0.035	0.550 ±0.089	0.454 ±0.039	0.597 ±0.049	**0.519** ±0.045	0.474 ±0.047	0.764 ±0.024	0.523 ±0.031	0.482 ±0.021	0.678 ±0.024	0.531 ±0.017
concat_row_center	0.494 ±0.058	0.659 ±0.025	0.547 ±0.068	0.470 ±0.034	0.606 ±0.021	0.511 ±0.051	0.469 ±0.065	0.771 ±0.031	0.508 ±0.049	0.478 ±0.025	0.679 ±0.023	0.522 ±0.012

**Table 7:** Classification performance by backbone model (5-fold cross-validation, mean ± SD). Bold indicates the best value in each column within each comparison block.

Method	N (Nuclear)			C (Cortical)			P (Posterior)			Average		
	F1	Acc	AUPRC	F1	Acc	AUPRC	F1	Acc	AUPRC	F1	Acc	AUPRC
*Backbone comparison (OCT only)*												
ResNet-34 (ImageNet)	0.415 ±0.020	0.570 ±0.024	0.442 ±0.016	0.448 ±0.034	0.587 ±0.037	0.465 ±0.033	0.438 ±0.041	0.722 ±0.039	0.461 ±0.047	0.434 ±0.021	0.626 ±0.027	0.456 ±0.025
ViT-L/16 (RETFound)	0.487 ±0.069	0.651 ±0.032	0.540 ±0.086	0.455 ±0.039	0.624 ±0.026	0.465 ±0.020	**0.517** ±0.029	0.757 ±0.043	**0.518** ±0.034	0.486 ±0.012	0.677 ±0.018	0.507 ±0.013
ViT-L/16 (ImageNet)	**0.504** ±0.069	**0.682** ±0.025	**0.550** ±0.060	**0.497** ±0.029	**0.633** ±0.020	**0.516** ±0.031	0.469 ±0.051	**0.765** ±0.023	0.511 ±0.031	**0.490** ±0.033	**0.693** ±0.009	**0.526** ±0.023
*Backbone comparison (Concatenation)*												
ResNet-34 (ImageNet)	**0.541** ±0.081	0.678 ±0.029	0.555 ±0.084	0.442 ±0.068	0.579 ±0.039	0.482 ±0.065	0.512 ±0.030	0.778 ±0.043	0.505 ±0.021	0.498 ±0.022	0.678 ±0.028	0.514 ±0.028
ViT-L/16 (RETFound)	0.524 ±0.060	0.681 ±0.044	**0.583** ±0.052	0.463 ±0.046	0.617 ±0.028	0.481 ±0.027	0.525 ±0.056	0.792 ±0.044	0.504 ±0.042	0.504 ±0.010	0.696 ±0.021	0.523 ±0.021
ViT-L/16 (ImageNet)	0.535 ±0.057	**0.688** ±0.034	0.569 ±0.073	**0.495** ±0.058	**0.623** ±0.049	**0.517** ±0.052	**0.563** ±0.056	**0.796** ±0.040	**0.585** ±0.036	**0.531** ±0.034	**0.702** ±0.031	**0.557** ±0.022

**Table 8:** Effect of Gaussian soft-label spread parameter σ on 5-fold cross-validated macro F1 scores (concatenation fusion, ViT-L/16).

σ	N-F1	C-F1	P-F1	Avg F1
0.3	0.545 ± 0.060	0.497 ± 0.041	0.579 ± 0.066	0.540
0.5	0.535 ± 0.057	0.495 ± 0.058	0.563 ± 0.056	0.531
0.7	0.347 ± 0.026	0.496 ± 0.072	0.357 ± 0.134	0.400

**Table 9:** Cross-modal attention performance by region setting (5-fold cross-validation, mean ± SD).

Region Setting	N-F1	C-F1	P-F1	Avg F1
Ours	0.535 ± 0.070	0.489 ± 0.032	0.551 ± 0.043	0.525 ± 0.035
Narrow	0.549 ± 0.058	0.465 ± 0.019	0.574 ± 0.079	0.529 ± 0.024
Wide	0.566 ± 0.074	0.497 ± 0.038	0.554 ± 0.069	0.539 ± 0.032

## Data Availability

The data presented in this study are available on request from the corresponding author due to institutional data-sharing policies.
